# Metabolic switches from quiescence to growth in synchronized *Saccharomyces cerevisiae*

**DOI:** 10.1007/s11306-019-1584-4

**Published:** 2019-08-29

**Authors:** Jinrui Zhang, Karla Martinez-Gomez, Elmar Heinzle, Sebastian Aljoscha Wahl

**Affiliations:** 1 Department of Biotechnology, Delft University of Technology, van der Maasweg 9, 2629 HZ Delft, The Netherlands; 2 Biochemical Engineering, Saarland University, Campus A 1.5, 66123 Saarbrücken, Germany

**Keywords:** *S. cerevisiae*, G0, G1, ^13^C flux analysis, Proteomics

## Abstract

**Introduction:**

The switch from quiescence (G0) into G1 and cell cycle progression critically depends on specific nutrients and metabolic capabilities. Conversely, metabolic networks are regulated by enzyme–metabolite interaction and transcriptional regulation that lead to flux modifications to support cell growth. How cells process and integrate environmental information into coordinated responses is challenging to analyse and not yet described quantitatively.

**Objectives:**

To quantitatively monitor the central carbon metabolism during G0 exit and the first 2 h after reentering the cell cycle from synchronized *Saccharomyces cerevisiae*.

**Methods:**

Dynamic tailored ^13^C metabolic flux analysis was used to observe the intracellular metabolite flux changes, and the metabolome and proteome were observed to identify regulatory mechanisms.

**Results:**

G0 cells responded immediately to an extracellular increase of glucose. The intracellular metabolic flux changed in time and specific events were observed. High fluxes into trehalose and glycogen synthesis were observed during the G0 exit. Both fluxes then decreased, reaching a minimum at t = 65 min. Here, storage degradation contributed significantly (i.e. 21%) to the glycolytic flux. In contrast to these changes, the glucose uptake rate remained constant after the G0 exit. The flux into the oxidative pentose phosphate pathway was highest (29-fold increase, 36.4% of the glucose uptake) at t = 65 min, while it was very low at other time points. The maximum flux seems to correlate with a late G1 state preparing for the S phase transition. In the G1/S phase (t = 87 min), anaplerotic reactions such as glyoxylate shunt increased. Protein results show that during this transition, proteins belonging to clusters related with ribosome biogenesis and assembly, and initiation transcription factors clusters were continuously synthetised.

**Conclusion:**

The intracellular flux distribution changes dynamically and these major rearrangements highlight the coordinate reorganization of metabolic flux to meet requirements for growth during different cell state.

**Electronic supplementary material:**

The online version of this article (10.1007/s11306-019-1584-4) contains supplementary material, which is available to authorized users.

## Introduction

All living cells adjust metabolism and cell growth to the nutrient availability in the environment. When conditions are unfavorable, cells enter quiescence (or G0), which is the most common cell state in natural environments (Gray et al. 2004). When nutrients become available, the quiescent cells are able to rapidly start metabolism, re-enter G1 and start the cell cycle progression. How cells process and integrate environmental information into coordinated physiological responses is still one of the most fundamental challenges in biology that is challenging to analyse and not yet described quantitatively (Ewald 2018). While multiple factors can regulate the transition between G0/G1, metabolic regulation was suggested to be the core determinant (Laporte et al. 2011). Different findings suggest that there is extensive cross-talk between different levels of the cell to coordinate metabolism and cell cycle progression, especially in cancer cells (Laporte et al. 2011; Kaplon et al. 2015; Ewald 2018). Despite recent advances, the detailed metabolic activity, i.e. the actual rate of metabolic reactions and pathways during the quiescence exit and subsequent cell cycle progression remain largely unknown.

Metabolic fluxes cannot be measured directly but have to be calculated from concentration and isotope-labeling experiments using computational tools (Abate et al. 2012; Klapa et al. 2003; Christensen et al. 2002). So far, only limited studies on this relevant level are available (Jouhten et al. 2012; van Winden et al. 2005; Ahn et al. 2017). Recent advances in ^13^C-based metabolic flux analysis (^13^C-MFA) now enable an accurate estimate of intracellular fluxes without assumptions about the activity or inactivity of parallel metabolic pathways (van Heerden et al. 2014; Schumacher and Wahl 2015; Ahn et al. 2017; Kleijn et al. 2005).

*Saccharomyces cerevisiae* is a proposed model organism to study metabolism as the yeast quiescence and proliferation switch is solely conditioned by nutrient availability (Daignan-Fornier and Sagot 2011). The molecular basis of the quiescence state and the re-entry into cell growth have been extensively studied and reviewed (Kaplon et al. 2015; Radonjic et al. 2005). Transcriptome analysis of quiescence exit in shake flask cultures showed an immediate upregulation of at least 2500 genes within 360 s (Radonjic et al. 2005). Distinct histone methylation and transcription profiles were observed for quiescence in yeast, which can rapidly be reversed and resume growth (Young et al. 2017). Laporte et al. found that the metabolic status and flux rather than cell cycle regulators are the principal triggers for the quiescence exit. The extracellular glucose concentration alone can trigger the first steps from G0 exit, but is not sufficient to trigger cell growth and proliferation. Glucose has to be metabolized at least into pyruvate to trigger mobilization of actin bodies (Laporte et al. 2011). Ewald et al. reported that at the G1/S transition, cyclin-dependent kinase 1 (CDK1) phosphorylates and activates the neutral trehalase (NTH1) to funnel trehalose into glycolysis (Ewald et al. 2016). A recent work shows that metabolic rates determine whether *S. cerevisiae* cells enter and successfully complete the cell division cycle (Litsios et al. 2018).

Most of these studies relied on shake flask or chemostat culture (Kresnowati et al. 2006; Radonjic et al. 2005). Here, to study the dynamic metabolic flux distributions through G0 exit and G0/G1 transition, the synchronization method proposed by Tu et al. (2005) was used. The approach is based on starvation and then limited, continuous feeding (D = 0.1 h^−1^) while no other chemicals are used allowing for a natural synchronization for about 10 h. The major metabolites and flux profiles (glycolysis, TCA, PPP, trehalose, glycogen, and glyoxylate shunt) were studied during G0 exit, the G1 phase and the initiation of S phase.

## Materials and methods

### Bioreactor cultivation and experiments set-up

**Fig. 1:**
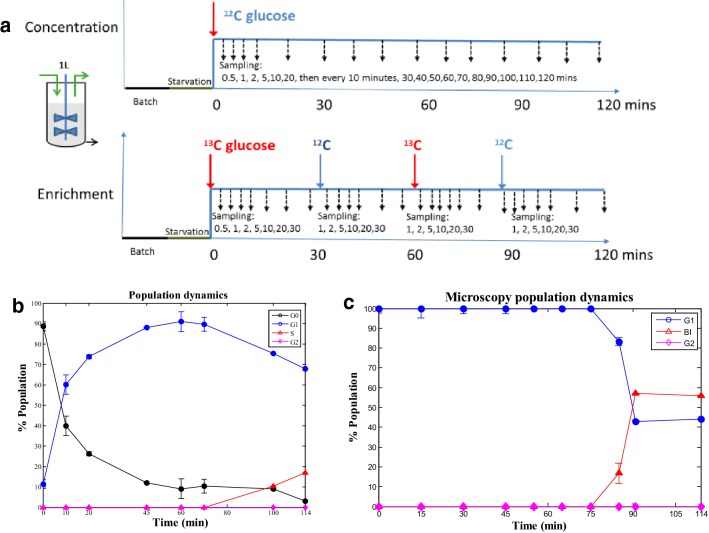
Experimental set-up and cell cycle state distribution. (**a**) Experimental set-up and sampling time points for concentration as well as labeling measurements in single and multi-switch experiments. Sampling time-points were denser after each switch in labeling to capture the fast as well as slow transitions in metabolites. (**b**) Cell cycle state based on FACS. (**c**) Budding index based on microscopic evaluation

Strain *S. cerevisiae* CEN.PK117-5D was obtained from the Centraalbureau van Schimmelcultures (Fungal Biodiversity Center, Utrecht, The Netherlands). A seed culture was performed using a shake flask culture aerobically at 200 rpm and 30 °C using 100 mL synthetic medium containing 20 g/L glucose (Verduyn et al. 1990). After overnight cultivation, the pre-culture was used to inoculate a 2 L bioreactor (Applikon Biotechnology B.V., Delft, The Netherlands) with 1 L working volume with 10 g/L glucose low-salt Verduyn minimal medium (Zhang et al. 2015). After batch growth, the culture was starved for 10 h to synchronize the cells at quiescence (G0), as previously described (Tu et al. 2005). After the starvation, the feed containing minimal medium with 10 g/L glucose was started and set at a dilution rate of 0.1 h^−1^ (Zhang et al. 2015). pH was kept constant at 5.0 by automatic addition of 2 M KOH. The temperature was controlled at 30 °C and the head space overpressure was kept at 0.3 bar. The aeration rate was 0.5 vvm and the stirrer speed was 600 rpm. Two same cultivation experiments were performed (Fig. 1a): (1) using ^12^C glucose in the continuous media for concentration measurements, and (2) using ^13^C uniformly labeled [U-^13^C_6_] glucose (Campro Scientific, Veenendaal, the Netherlands) in the continuous media for ^13^C tracing, ^13^C and ^12^C glucose medium were switched every 30 min to enable flux identification for 120 min. The biomass dry weight concentration was measured as described (Zhang et al. 2015) and also monitored by the BE2100 biomass sensor, an on-line optical density measurement (BugLab LLC Danville, CA) to obtain short-term growth rate estimation. The biomass concentration and growth rate were interpolated using a simple black box model and parameter optimization (Fig. S4).

1 $$\frac{{dc_{X} }}{dt} = \mu c_{X} - Dc_{X} .$$

### Flow-cytometric analysis of DNA content

Flow cytometric analysis was used for determining DNA content (FL2-A histogram), size by forward-scattered light height (FSC-H) and internal complexity side-scattered light height (SSC-H) respectively, to determine G0 population and cell cycle stage of the cells as previous reported (Delobel and Tesnière 2014). Populations from 0, 10, 20, 25, 45, 60, 70, 100 and 114 min were evaluated. About 4 × 10^6^–2 × 10^7^ cells (approximately 50 μL of culture) were spun down in Eppendorf tubes for 5 s at 14,000 ×*g*. Cells were resuspended in 1 mL deionized water and spun down. The fluid was poured off, and cells were resuspended in the remaining traces of water by vortexing (this prevents excessive clumping during the following fixation). The cells were fixed in 70% ethanol. Samples were stored at 4 °C overnight. Stored samples were washed three times with 0.5 M sodium citrate, pH 7. 0. 25 mL RNase solution (10 mg/mL) was added and samples were incubated for 1 h at 37 °C. Cells were centrifuged for 2 min at 14,000 ×*g* and resuspended in staining buffer [0.05 M sodium citrate (pH 7), 0.01 M NaCl, 0.5% Nonidet-P40 and 16 mg/mL propidium iodide (PI)]. Samples were incubated for at least 2 h at 4 °C. Finally, cells were pelleted and resuspended in 1.5 mL EDTA solution (10 mM, pH 5), sonicated for 15 s and analysed by flow cytometry, measuring and recording 25,000 or 50,000 events.

Fluorescence of the PI-stained cells was measured using the FL2 channel using FACScan (Becton–Dickinson Immuno cytometry Systems, Saarbrücken, Germany). FL2-A versus FSC-H plots were analysed using Flowjow software to reveal the percentage of cells in G0, G1, and S (Fig. S1). FACSCalibur parameters: the fluorescence emission maximum for DNA-bound PI is about 615–620 nm. When excited by a 488 nm laser, PI can therefore be detected in both, the red/orange emission channel (FL2) tandem dye detection as well as the red emission channel (L3). Data acquisition and analysis were done by using CellQuest software R and Flojow (Zhang and Siede 2004).

The area for G0, G1, S and G2/M cells stained with PI was reported to be in the scatter area as shown in Fig. S1. For determining different cell stages populations 10 h starvation sample, 10 min sample and samples from various fermentation time points 60, 90, 100, 125 were compared to gate populations. FL2-A versus FSC-H plots showed that population 10 min changed its scatter area as compared to the 10-h starvation samples (Fig. S1a1, a2). Also the 10 min samples had a different side-scattered fluorescence (SSC) distribution as compared to the 10 h sample (Fig. S1c1, c2). Distribution of forward-scattered light (FSC) fluorescence which is proportional to the cell-surface area, showed no differences in the plot area for all samples. These results together indicate that 10 h starvation (G0) samples had smaller nuclei as compared to the samples of different sample points but showed no difference in cell size. These results agree with the report of Laporte et al. that G0 phase leads to chromatin condensation (Laporte et al. 2016).

### Microscopic evaluation of DNA content

Microscopy was used to determine budding index (BI) and nuclei localization (Fig. 1c). This information was used to discriminate population in S phase using flow cytometry which appears to increase from 100 min onwards. For mounting solution, 100 mg *p*-phenylenediamine was dissolved in 10 mL PBS, the pH was adjusted to above 8.0 with 0.5 M sodium carbonate buffer (pH 9.0), and the volume was brought to 100 mL with glycerol. From a 1 mg/mL stock solution in water, 4′,6-diamidino-2-phenylindole (DAPI) was added to 50 ng/mL. After mixing, the solution was stored at − 20 °C. Solutions that have turned brown were discarded. Calcofluor white stain was prepared according to provider instructions (Sigma-Aldrich, Munich, Germany), mixing it in equal volume with KOH 10%. Cells fixed in ethanol were washed three times with deionized water and finally suspended in 1 mL, from this cell solution 10 µL was left in a slide and incubated at 37 °C to dry. The slides were analysed by adding one drop of mounting solution and one drop of calcofluor and analysed in microscope Olympus 100^R^.

### Rapid sampling, metabolites, mass isotopomer extraction and analysis

The sampling times for concentration measurements were at 0.5, 1, 2, 5, 10, 20, 30, 40, 50, 60, 70, 80, 90, 100, 110, 120 min. For enrichment measurement, the sampling was more intensive to capture the rapid changes between the switch of ^13^C and ^12^C glucose (Fig. 1a). The sampling, quenching, extraction and analysis of the metabolites were performed similar to the methods described earlier (Zhang et al. 2015). In brief, 120 μL of ^13^C cell extract was added an internal standard for concentration measurements samples based on the isotopic dilution mass spectrometry method. The intracellular metabolites were analysed by GC–MS or LC–MS/MS. The procedure was similar for mass isotopomer enrichment samples with the exception that no ^13^C cell extract was added. The G0 samples were measured in triplicate. During the dynamic experiments after the feeding, only single samples could be taken at each time point, and time series measurements contain a certain level of redundancy because of the dense sampling time.

### Measurement of protein levels

Proteomics samples were taken at 0, 30, 60, 90, 120 min, and steady-state. U-^13^C labeled *S. cerevisiae* biomass was used as internal standard for relative protein quantification (Cueto-Rojas et al. 2017). Protein quantification was restricted to peptides with Mascot score ≥ 25. A protein is considered differently expressed when the concentration is at least 20% higher or lower compared to the reference or other samples. The proteins that were found in one or a few conditions but not in the other conditions are considered “unique” proteins.

### Metabolic flux analysis

Intracellular fluxes were estimated based on the metabolic network model specified in Table S1. The model consists of 41 reactions belonging to glycolysis, PPP, trehalose and glycogen pathway, TCA cycle, glyoxylate cycle, several amino acids, and 9 biomass reactions. Biomass formation is defined by a set of fluxes originating from G6P, F6P, PG3, G3P, PEP, E4P, Rib5P, trehalose, and glycogen, which serve as molecular precursors for biomass formation. By combining the concentration and enrichment measurements, dynamic ^13^C-MFA using a hybrid modelling approach with piecewise affine (PWA) flux approximations was applied (Abate et al. 2012, Schumacher and Wahl 2015). The breakpoints (number and times) were determined using an optimization approach to best capture the response of the measured metabolites (Schumacher and Wahl 2015). With an initial guess for the (independent) fluxes at each breakpoint, the estimation of fluxes was performed based on least-squares optimization to fit both concentration and labeling enrichment measurements. The image was prepared using the visualization software Omix (Droste et al. 2011).

### Functional enrichment analysis

GO enrichment analysis was performed by the PANTHER (protein annotation through evolutionary relationship) classification system (Mi et al. 2016) based on four given ontology association (biological process, pathway, molecular function and protein class). Cluster analysis was performed using the GenePattern expression data analysis package (Reich et al. 2006).

## Results and discussion

### Cell synchronization and population distribution

A *S. cerevisiae* culture was grown in mineral media without yeast extract and driven into G0 state by carbon starvation for 10 h (Tu et al. 2005). Then the culture was supplied with fresh medium at a dilution rate of D = 0.1 h^−1^ (Fig. 1a). G0 and G1 population was discriminated using fluorescence-activated cell sorting (FACS). After 10 h starvation 89% of the population was in G0 and 11% was in G1. Ten minutes after initiating the continuous feed the G0 population dropped to 40% and G1 increased to 60% (Figs. 1b, c, S1). G1 population appeared to reach its maximum from 45 min onwards (90%). DAPI-calcofluor (4′,6-diamidino-2-phenylindole)-microscopy was used to analyse BI and nuclei localization to discriminate population in S phase (Figs. 1c, S2). BI appeared to increase only after 75 min, and BI index increased from 16 to 56% from 85 to 114 min (Fig. 1c). This information was used to discriminate the population in S phase using FACS which appeared to increase from 100 min onwards. Thus, during the monitored 2 h of cell cycle progression, the phases of G0/G1 transition, G1, and G1/S transition could be observed. The partial synchronization was similar to the reported synchronized yeast cell population through centrifugal elutriation and other synchronization methods (Costenoble et al. 2007; Schmidt 2014). The ${\text{p}}_{{{\text{O}}_{2} }}$ and offgas O_2_ characteristic comparisons and the correlation plot of the two ${\text{p}}_{{{\text{O}}_{2} }}$ profiles of the different cultivations supports that the ^12^C and ^13^C fed cultures were very comparable (Fig. S3). The biomass concentration was nearly constant. Some decrease (5%) was observed between 40 and 80 min. Then an increase was observed after 80 min (Fig. S4). There was no detectable secretion of metabolic byproducts, like ethanol or glycerol. Therefore, the biomass constituents were fixed in the biomass reaction.

### Estimation of intracellular fluxes during synchronization

Dynamic ^13^C-MFA using PWA flux approximations was applied to determine intracellular fluxes based on measured metabolite concentrations, enrichments, and the metabolic network model specified in Table S1. The experiment was designed to enable a sensitive monitoring of the glucose uptake rate, i.e. no significant accumulation of substrate in the broth (feeding at D = 0.1 h^−1^). Furthermore, a feed switching between ^13^C_6_ and unlabeled glucose every 30 min was used to generate labeling transients during 120 min and allow for a longer experiment compared to a classical wash-in experiment (Fig. 1a). The sampling time points were designed to capture the enrichment gradients during the switches between ^13^C and unlabeled glucose of fast and slow turnover pools.

**Fig. 2:**
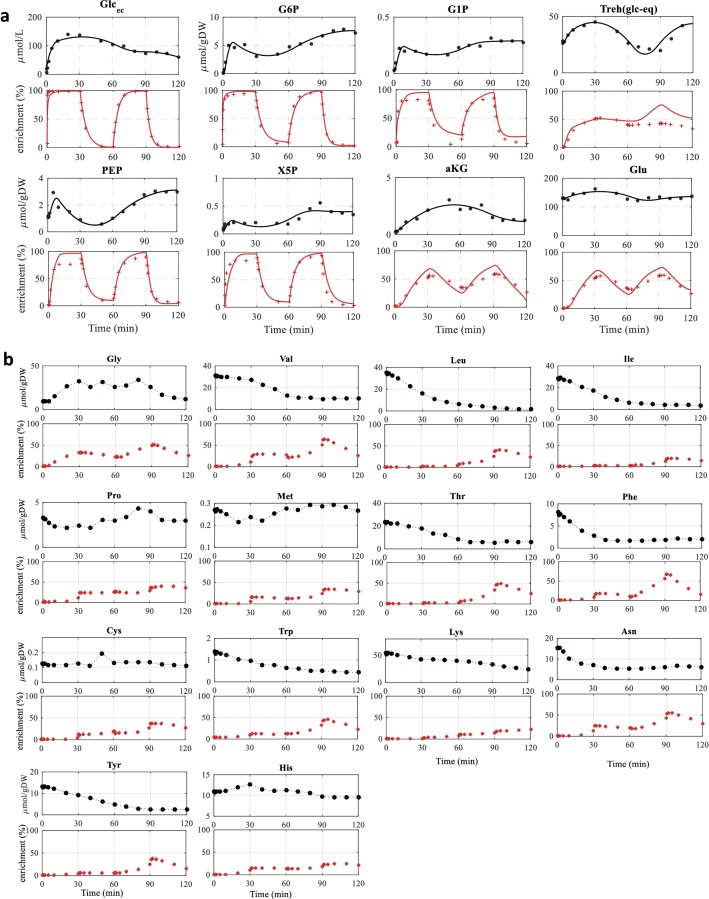
Measurements of representative intracellular metabolite concentrations (black dot), enrichments (red cross). If included in the PWA model, the result from the simulation is shown as line. (**a**) metabolites and (**b**) amino acid. Additional metabolite concentrations, enrichments and simulations are shown in Fig. S7

The best fit obtained for the intracellular concentrations as well as labeling enrichments is shown in Figs. 2 and S7. For some TCA metabolites, i.e. citrate (Cit), iso-citrate, and glyoxylate, the simulation could not reproduce the enrichments, which could be due to compartmentation (not included in the metabolic network model). The standard deviation of the estimated metabolic flux was calculated using (linearized) error propagation: the equation system was linearized at each measurement time point by differential differentiation. With the assumed measurement errors, the standard deviation of the flux was determined. Details of the calculation approach can be found in Wahl et al. (2008). Please note that these standard deviations are a lower bound, assuming that there are no errors in the concentration measurements (so for example the uptake rate has no standard deviation). The estimation of anaplerotic reactions, especially for compartmentalized organisms like yeast has high standard deviations. Consequently, the observed correlation is potentially influenced by these inaccuracies. All the standard deviations are shown in the supplemental Table S2.

**Fig. 3:**
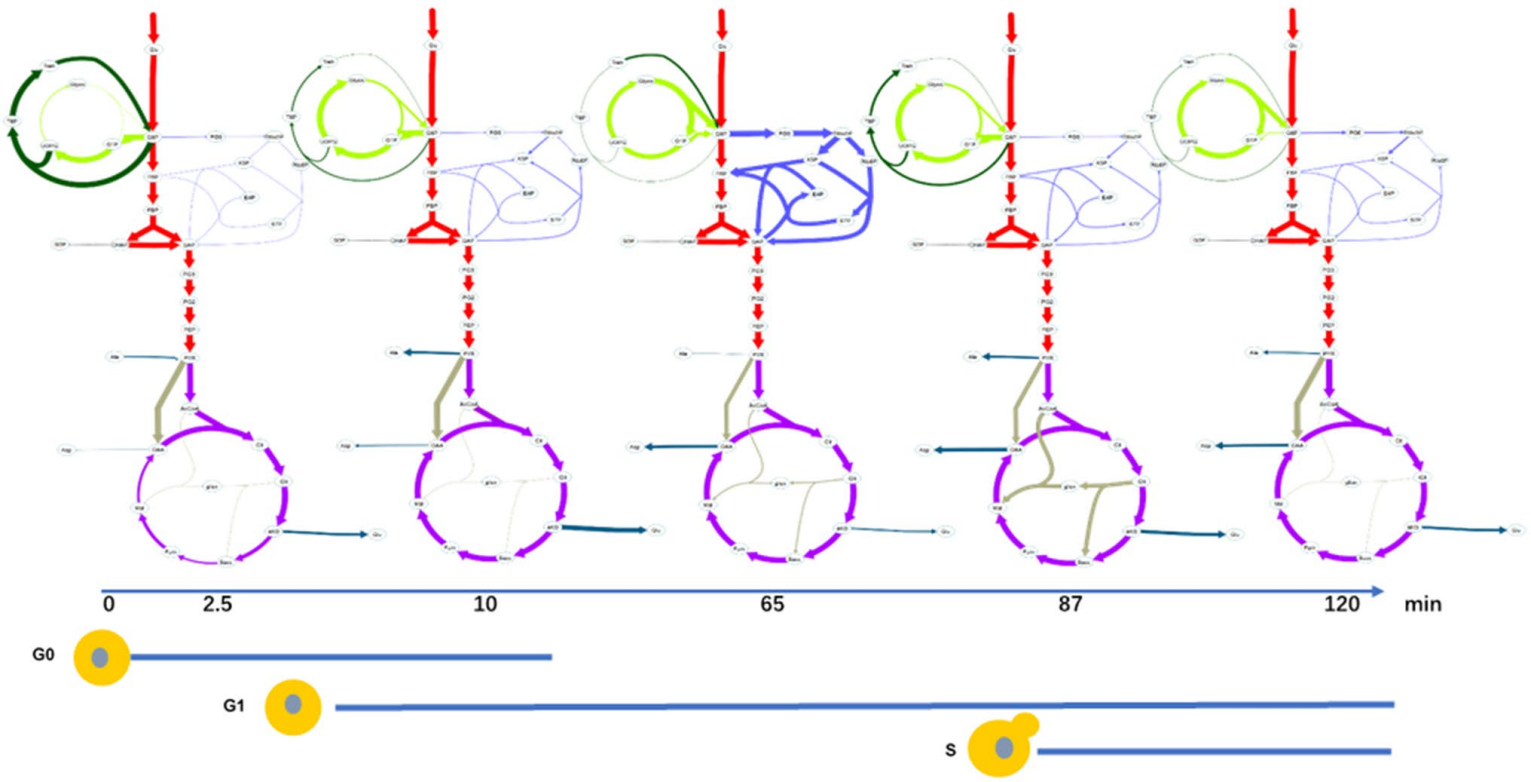
Snapshots of the estimated metabolic fluxes aligned with the predominantly determined cell cycle states. Fluxes colors: red, glycolysis; blue, PPP; purple, TCA; light green, glycogen pathway; dark green, trehalose pathway; grey, glyoxylate; dark blue, exchange with amino acids. Flux maps with more details can be found in Fig. S8, flux values are documented in Table S2

The flux function breakpoints were placed at 0, 2.5, 10, 65, 87 and 120 min. The estimated metabolic fluxes at the breakpoints are depicted in Fig. 3. Graphs with more details can be found in Fig. S8 and the flux values are listed in Table S2.

**Fig. 4:**
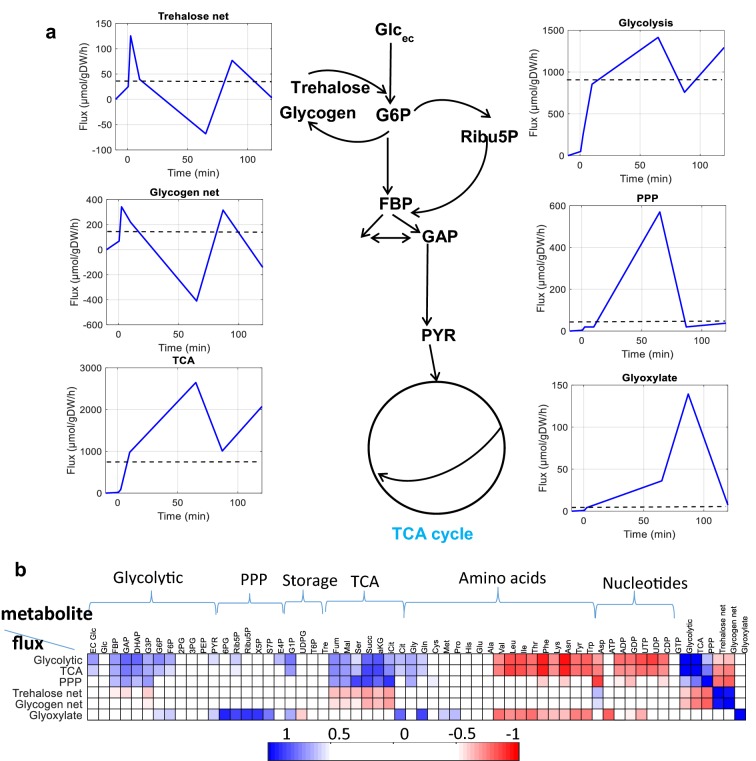
Metabolic fluxes dynamics in glycolysis, TCA, PPP, trehalose, glycogen, and glyoxylate pathways during the transition (**a**) and correlation with metabolites concentrations (**b**). Correlation values between − 0.5 and 0.5 are shown in white

To elucidate putative influencing metabolites, correlations between pathway fluxes and metabolite concentrations were calculated (Fig. 4). Here, the glycolytic and TCA fluxes were correlated with each other (r = 0.95), and FBP, GAP, DHAP, and most TCA metabolites were positively correlated with the glycolytic, TCA fluxes (r = 0.6–0.8). In contrast to this correlation, the trehalose and glycogen fluxes were correlated with each other (r = 0.91), but not with the involved metabolites and precursors. The oxidative PPP flux did not correlate with other fluxes and also no correlation with the metabolites of the PPP were found.

The enrichment patterns were quite different among the amino acids (Fig. 2b). For many amino acids which decreased after the feed till 120 min, the corresponding enrichment was much lower and slower compared to central carbon metabolism. This could suggest a delayed increase in amino acids synthesis. Exceptions are Ala, Ser, Asp and Glu that were faster enriched and deenriched following the ^13^C and ^12^C switches (Fig. S7). This could be explained by exchange fluxes of these amino acids with the respective precursors in central carbon metabolism, which were in the range of 1–2% of the glucose uptake rate (Table S2).

Therefore, the response in fluxes and metabolites are discussed below based on the time-intervals between break points and the observed cell state 0–10 min (G0/G1 entry), 10–87 min (G1), and 87–120 min (late G1 and S phase transition).

### Metabolite dynamics during G0/G1/S transition

The energy charge is considered an important physiological parameter for the cellular state. Here we observe that nucleotide levels of G0 cells were comparable to the reference steady-state condition (D = 0.1 h^−1^) (Fig. S6a), i.e. the ATP concentration (10.5 vs. 12.6 µmol/g DW) and also the energy charge (0.91 vs. 0.87) (Fig. S7b). Furthermore, the energy metabolites were nearly constant during the transitions. Thus, although the extracellular glucose uptake rate changed significantly there is homeostasis in the energy metabolites.

In G0 cells, 28% of the 54 measured intracellular metabolites showed a higher (1.2- to 62-fold) concentration compared to the reference condition (D = 0.1 h^−1^). 50% of the metabolites were lower (1.2- to 100-fold change) (Fig. S6a). Especially: (1) upper glycolysis had significantly reduced concentrations while TCA cycle intermediates remained comparable to growing cells, (2) some amino acids (Ser, Asn, Thr, Trp, Gly, Lys, Phe, Ile, Tyr and Leu) were increased more than 8-fold. The pathway specific increases or decreases suggest a coordinated regulation of these metabolites. Especially, the increase in specific amino acids like Trp or Phe that require ATP during synthesis supports a coordinated behavior.

Also, during the G0/G1 transition, coordinated changes are observed. Especially, three major groups of metabolites are identified by hierarchical clustering (Fig. S6b): (1) concentration low in G0, increased to a maximum around 90–120 min (later G1/S phase), (2) an increase till a maximum around 50 min (middle G1 phase) was reached, after that a decrease, (3) the highest concentration was observed in G0 and then decreased till 120 min.

The glycogen pool was almost depleted (~ 0 µmol/g DW) after 10 h of starvation while trehalose was one of the largest intracellular pools (26.5 µmol/g DW, 16% of steady-state value) (Fig. 2a). With initiation of the feeding, both glycogen and trehalose were first built up (within 30 min), then started to decrease and increased again between 65 and 120 min.

### Flux dynamics during G0/G1/S transition

#### t = 0–10 min (extracellular glucose uptake triggers G0 exit)

In the first 10 min after feeding, the extracellular glucose concentration increased from 3.7 to 446 μmol/L (Fig. S5) which was higher compared to the later steady-state concentration. The glucose uptake rate increased from 0 to ~ 1.4 mmol/g/h within 10 min, consuming nearly all glucose fed to the bioreactor (Fig. S5). While the glucose uptake rate was nearly constant after 10 min, the extracellular glucose concentration still increased until about 20 min. This effect is more visible in Fig. S5c, showing the uptake rate as a function of the extracellular concentration. First, the glucose uptake rate increased proportionally with the glucose concentration. Later the uptake was constant while the glucose concentration decreased. This suggests that there are adjustments in the glucose transport capabilities, possibly due to the synthesis of transporters with different affinity and uptake capacity over the fermentation time course.

Looking at the intracellular space, it can be observed that the metabolic flux during the first 2.5 min was directed towards glycogen synthesis (44% of the uptake rate, 343 μmol/g DW/h), trehalose synthesis (16%, 125 μmol/g DW/h) and glycolysis (37%, 285 μmol/g DW/h). Only 3% (20 μmol/g DW/h) was directed to oxPPP (Figs. 3, S8). The TCA flux increased as the glycolytic flux increased, and the glyoxylate flux was almost 0.

At 10 min, the incoming glucose was directed more towards glycolysis (77%, 939 μmol/g DW/h), and much less to trehalose (3.2%, 40 μmol/g DW/h), glycogen (18%, 220 μmol/g DW/h) and oxPPP (1.6%, 20 μmol/g DW/h) (Fig. 3, Fig. S8 and Table S2). The TCA flux increased similar to the glycolytic flux, and the glyoxylate flux was still low, and less than 1% of the TCA flux.

With the increase of extracellular glucose and uptake, a switch in cell cycle state was observed, i.e. 60% of the population entered G1 within 10 min. Given the short time span, it is assumed that the G0 exit was mainly triggered by the changed extracellular conditions and the metabolic activity rather than transcriptional changes. Similarly, the metabolic flux was most probably regulated by non-hierarchical mechanisms as translation and transcription.

#### t = 10–87 min (metabolism drives cell growth: cell status)

At t = 65 min both trehalose and glycogen were degraded to G6P (total 21% of the glucose uptake flux, 3% from trehalose and 18% from glycogen). The glycolytic and TCA fluxes reached the maximum (Fig. 3). At the same time the flux entering the oxPPP increased significantly to 570 μmol/g DW/h, corresponding to an oxPPP/glycolysis split ratio of 36.4%.

This value is in agreement with findings of van Winden et al. (2005), reporting split ratios between 5 and 52% (van Winden et al. 2005). While no S phase cells were found yet, the maximum oxPPP/glycolysis split ratio suggests the main aim of the oxPPP flux at this time was to generate enough nucleic acid precursors and NADPH to support cell cycle progression, which would be in accordance with the increased production of NADPH during the ‘reductive/charging phase’ of the metabolic cycle (G1/S transition), as suggested earlier (Cai and Tu 2012).

#### t = 87–120 min (cell cycle progression drives metabolism)

At t = 87 min, some flux was again directed towards glycogen synthesis (25% of the glucose uptake flux, 316 μmol/g DW/h) as well as trehalose (6%, 77 μmol/g DW/h). Significantly decreased is the flux into the oxPPP (1.5%, Fig. 3). Additionally, the glycolytic flux and TCA flux decreased significantly. Notably, the flux to the glyoxylate cycle increased significantly and reached the maximum at this point, about 14% of the TCA flux.

At 120 min, most of the flux was directed to glycolysis (88%, 1381 μmol/g DW/h) and glycogen was again degraded, generating additional flux into G6P (9%, 141 μmol/g DW/h) (Fig. 3). The flux through the oxPPP pathway (2%, 38 μmol/g DW/h) was much lower than at 65 min. The flux through the trehalose pathway (0.2%, 3 μmol/g DW/h) was almost neglectable. The TCA flux also increased, similar to the glycolytic flux, but the glyoxylate flux decreased to 0.

### Protein dynamics during G0/G1/S transition

Proteomics samples were taken at 0, 30, 60, 90, 120 min, and the steady-state. 1030 Proteins were identified and quantified using at least 2 unique peptides and another 800 proteins were identified with 1 unique peptide since only one unique peptide is available for these proteins. Comparing G0 cell to the steady-state, the following observations of the relative changes were made (Table S2):

1. No significant changes for most central metabolic enzymes: PGI (122%), PFK1 (115%), PFK2 (109%), FBA (84%), TPI (100%), PGK (90%), PGM (98%), GPM (94%), HXK2 (106%) with an average standard deviation of 20%. Most of those reactions are supposed to be equilibrium reactions (Canelas et al. 2011). The significantly decreased enzymes were: HXK1 (41%), GND1 (55%), TDH1 (62%), ENO1 (48%), GDH1 (26%), and these reactions are normally far from equilibrium.
2. Most gluconeogenic, TCA and glyoxylate cycle enzymes had higher levels, e.g. FBP1, ICL1, MLS1. Transporters and enzymes involved in growth on non-sugar carbon substrates were expressed.
3. Heat shock and stress resistant proteins were increased.

Almost all those unique proteins (that were found in one condition but were too low to be detected in other samples) or significantly changed proteins in our conditions agreed with the reported proteomics of stationary/G0 phase cells (Kumar and Srivastava 2016). Some examples are: HSP12, SOD1, and HXT5.

**Fig. 5:**
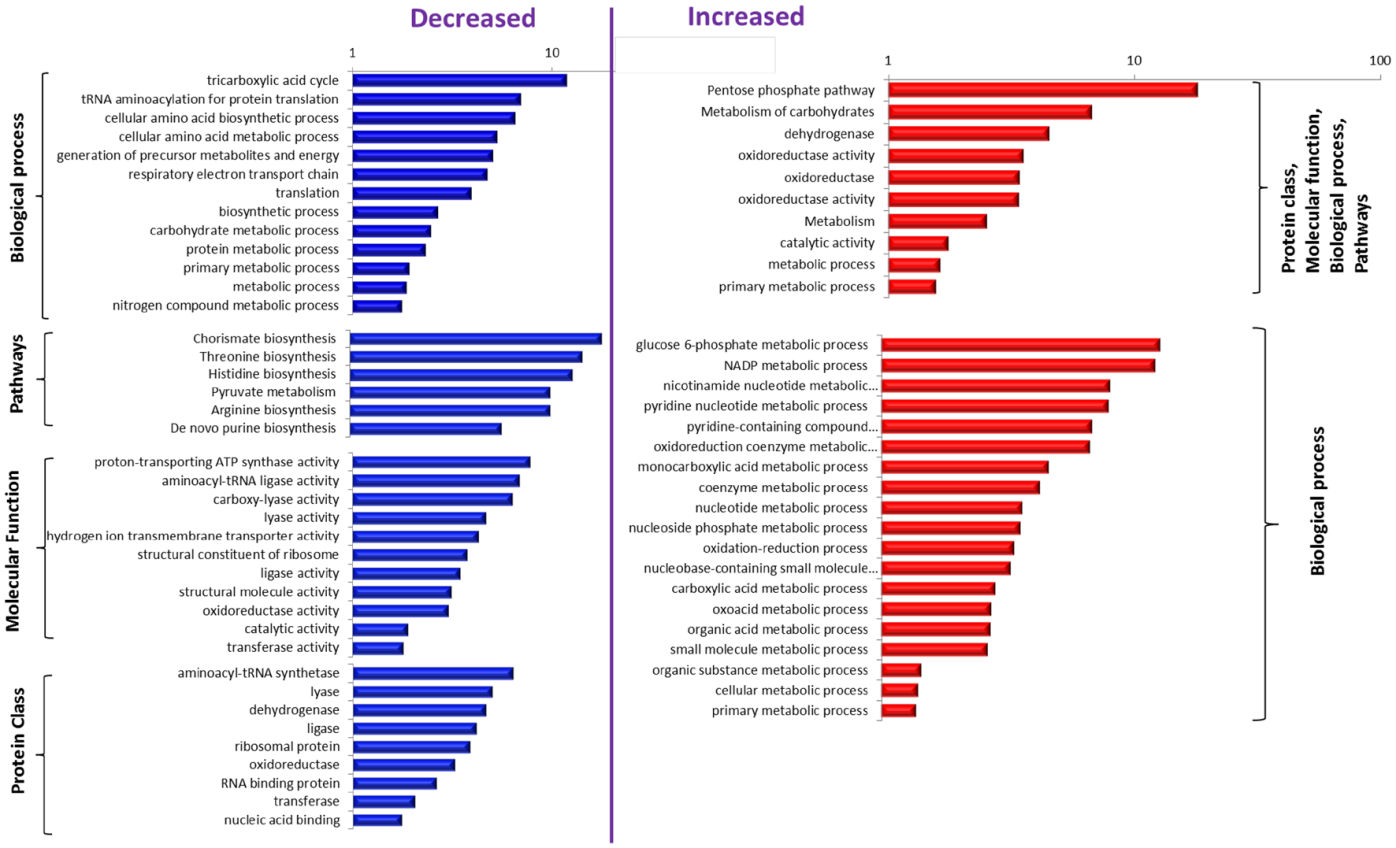
Enriched functional categories of decreased and increased proteins during the transition. Performed by the PANTHER (protein annotation through evolutionary relationship) classification system based on four ontology association (biological process, pathway, molecular function, and protein class). The particular functional categories were shown as significantly enriched if P < 0.05. The axis shows the log of fractional difference (observed vs. expected)

Key functional categories of decreased and increased proteins during this transition were classified using PANTHER (Fig. 5). Based on the specific expression during this transition, these proteins could be critical for reaching (and leaving) the G0 state, and G0/G1/S transition.

#### t = 0–65 min

The metabolic activity during the first 2.5 min after glucose feeding was a result of the proteins already present in G0 and activated by the presence and metabolism of glucose. Although there was a probability of new synthetized proteins during the first 2.5 min, the apparatus of replication might be scarce and the translation apparatus might be not fully habilitated as further protein analysis suggests. As a result the flux at this phase is the consequence of metabolic regulation. The presence of glucose and increase in the metabolite concentration can be sensed by regulators to initiate the first transcriptional remodeling. After 10 min fluxes are the result of the reshape of the metabolism by hierarchical and metabolic regulation; in other words, because of new proteins synthetized and/or by interactions of enzymes with substrates, products and allosteric effectors. The proteins of those significantly related pathways were sorted and grouped in the heat map according to metabolic pathways (Fig. S9). In general, the protein levels of the TCA cycle, de novo purine biosynthesis and chorismate pathways were higher than at steady-state, and the enzymes of glycolysis, storage, and PP pathways were lower.

The proteins with more important changes in concentration were derived from protein measurement at t = 30 min. These proteins belong to nucleotide phosphate metabolic process, nucleotide metabolic process, cellular amino acid metabolic process, polysaccharide metabolic process, cellular polysaccharide metabolic process, and carbohydrate metabolic process.

1. Glucose metabolism, amino sugar metabolism, fructose and mannose metabolism, N-glycan biosynthesis: HXK1, UGP1, GPH1 and VAN1. Glycerophospholipid-glycerolipid metabolism: GUT1, especially GPP1, GPT2 and ALD4 increased its concentration.
2. Amino acids pathways: proteins that belong to glutamate metabolism, arginine biosynthesis and degradation and proline biosynthesis were highly produced. Arginine and proline and metabolism were highly induced during the transition, which agrees with the doubling of the flux from α-ketoglutarate (aKG) at t = 10 min.
3. RNA polymerase biogenesis, ribosome biogenesis, ribosome exportation and spliceosome: RNA polymerase subunits, corresponding to the RNA polymerase activity (RNA polymerase I and II) for transcription of ribosomes and mRNA and snRNA, increased their production from 0 to 30 min. Ribosome subunits synthesis RPL12A, RPS31, RPL37A, RPL38 and RPL29 were also induced during transition. Transcription factors HCR1 and TIF5 involved with termination/recycling phases of translation were also peaked in this period. PRP43, spliceosome, a subunit involved in the release of mature mRNA was beginning its transcription during this phase. All these processes coincide with the G1 entrance and cell growth development.

Several rearrangements of fluxes at the level of PPP occurred at 65 min (G1 phase). The change at the level of PPP proteins coincides with a drop in concentration in most of the rest of the proteins. The drop in concentration could be related to an enhanced volume of the cells due to growth. GND1 concentration enhanced at this phase, which agrees with the turn off of the production of Rib5P. This high rearrangement at the level of PPP is for enhancing the level of nucleotides and NADPH necessary for RNA transcription that is enhancing demand during late G1. It has been reported that an increase in NADPH demand to 22 times the anabolic requirement for NADPH was accompanied by the intracellular accumulation of PPP metabolites consistent with an increase in flux through this pathway. NADPH demand agreed with NCP1 concentration peak. NADPH–cytochrome P540 reductase is a protein that catalyzes the transfer of electrons from NADPH to the cytochrome P450 protein, which is required to activate oxygen for the oxidation reactions. In addition GND1, UGA1, MAE1 and HAS1 belong to the oxidative stress pathway. Reactive oxygen species (ROS) can be generated as a result of an enhanced respiration which could signal the initiation of S phase in a later stage. It has been reported that increase in the ROS steady-state level is required for entry into S phase, i.e. an increase in endogenous ROS levels in fibroblasts is necessary for cell cycle progression through the G1 phase of the cell cycle (Havens et al. 2006). In G1, ROS stimulate mitogenic pathways that control the activity of cyclin-dependent kinases (CDKs) and phosphorylation of the retinoblastoma protein (pRB), thereby regulating S phase entry. In summary, it seems that at this time point (t = 65 min), G1/S transition occurred, which was 15 min before detecting budding clearly in a percentage of the cell population.

#### t = 87–120 min

According to the proteomics data, process of degradation of fatty acids could be enhanced at this point. Interestingly ALD4 decreased its concentration probably to allow acetyl-CoA (AcCoA) be metabolized by the glyoxylate cycle. PRP43 (spliceosome), GDH1 (NADPH generation) and NDE (NADH) peaked at this time point, and NADH and NADPH pathways were also highly active. At 120 min, a peak in concentration in UGP1 could be related with a high influx to glycogen build up and burn. In addition, GND1, an isoenzyme of the oxidative pentose phosphate reached a peak at 120 min which doubles the flux at 87 min. HXK1 peaked at this stage probably to phosphorylate income glucose or free glucose. Interestingly, one subunit of the fatty acid oxidation FAA1 also peaked at this stage, in addition ALD4 peaked at this point probably to avoid the production of by-products and reroute AcCoA towards TCA. Glutamate synthesis and degradation (UGA1 and CAR2) can be highly active during S phase. Interestingly a multifactor translation initiation factor (TIF5) peaked at this phase. In addition, RPL38, one subunit of the ribosome peaked at this stage.

## Conclusion

Using current fluxomics and metabolomics approaches, the metabolite and flux pattern in *S. cerevisiae* during G0 exiting, re-entering G1 and the initiation of the S phase was monitored. Additionally, protein levels were measured during the transition. The used experimental setup consisted of two independent bioreactor cultivations, operated under the same conditions. Based on this biological redundancy, together with the applied dense time series measurements, we are convinced that the obtained results are highly reliable and reproducible.

G0 cells responded very quickly to increased extracellular glucose availability and rapidly entered G1 (88% of the population) in 25 min. The glucose uptake rate increased from 0 to 1452 μmol/g DW/h within 10 min. The short time span of the first transition suggests that the G0 exit was mainly triggered by the changed extracellular conditions (glucose) and metabolic activity. In later phases the contrary was observed, i.e. intracellular fluxes did change while the extracellular environment (glucose concentration) was constant. This suggests that after 10 min, intracellular mechanisms like cell cycle dependent regulation rather than extracellular stimuli influenced the metabolic fluxes.

The flux distribution changed dynamically and specific events were observed during the transition. Especially,

1. Storage metabolism was highly dynamic: the fluxes to trehalose and glycogen were highest during G0 exit.
2. Both trehalose and glycogen then decreased to the minimum at t = 65 min, and degradation of storage compounds contributed significantly to the glycolytic flux (21%).
3. A major rearrangement occurs at the level of PPP at t = 65 min, and oxPPP/glycolysis split ratio reached a maximum of 36.4%. In all other phases the oxPPP flux was much lower (< 10%). This rearrangement occurred in late G1 in which G1/S transition seems to occur.
4. Anaplerotic reactions such as glyoxylate shunt increased at t = 87 min (initiation of S phase).

The model used in this work did not consider different compartments. This simplification might bias the results obtained for the TCA cycle fluxes, i.e. when there would be significantly different metabolism in the mitochondria and in the cytosol (Wahl et al. 2017). However, because the ^13^C model does not balance cofactors, there is no impact of TCA cycle flux values on glycolysis or other pathways.

During the whole transition, significantly increased or decreased proteins were identified. Overproduced proteins in G0 were those belonging to gluconeogenesis, TCA activity proteins, AcCoA recycling and β-oxidation, carbon and oxygen stress response related. During G0/G1 transition proteins belonging to clusters related with ribosome biogenesis and assembly, and initiation transcription factors clusters were continuously synthetized. In this work we enlight the flux rearrangements occurring in G0 cells after glucose presence and the subsequent rearrangements to have a fully mature G1 cell capable to commit S phase are shown.

## Electronic supplementary material

Below is the link to the electronic supplementary material.

Supplementary material 1 (DOCX 3333 kb)

## Metabolites

aKG: α-Ketoglutarate
AcCoA: Acetyl-CoA
Cit: Citrate
DHAP: Dihydroxy acetone phosphate
E4P: Erythrose-4-phosphate
F6P: Fructose-6-phosphate
FBP: Fructose-1,6-bis-phosphate
Fum: Fumarate
G1P: Glucose-1-phosphate
G3P: Glycerol 3-phosphate
G6P: Glucose-6-phosphate
GAP: Glyceraldehyde-3-phosphate
Glc: Glucose
Glyco: Glycogen
Glyox: Glyoxylate
iCit: Iso-citrate
Mal: Malate
OAA: Oxaloacetate
PEP: Phospho-enol-pyruvate
PG2: 2-Phosphoglycerate
PG3: 3-Phosphoglycerate
PG6: 6-Phosphogluconate
Pyr: Pyruvate
Rib5P: Ribose-5-phosphate
Ribu5P: Ribulose-5-phosphate
S7P: Sedoheptulose-7-phosphate
Succ: Succinate
Treh: Trehalose
T6P: Trehalose-6-phosphate
UDPG: UDP-glucose
X5P: Xylulose-5-phosphate

## Amino acids

Ala: Alanine
Asn: Asparagine
Asp: Aspartate
Cys: Cysteine
Gln: Glutamine
Glu: Glutamate
Gly: Glycine
His: Histidine
Ile: Iso-leucine
Leu: Leucine
Lys: Lysine
Met: Methionine
Phe: Phenylalanine
Pro: Proline
Ser: Serine
Thr: Threonine
Trp: Tryptophan
Tyr: Tyrosine
Val: Valine

## Enzymes

HXK1, HXK2: Hexokinase isoenzyme 1, 2
GLK1: Glucokinase 1
FBP1: Fructose-bisphosphatase 1
MAE1: Malic enzyme
GND1: Phosphogluconate dehydrogenase
TDH1: Glyceraldehyde-3-phosphate dehydrogenase
ICL1: Iso-citrate lyase
ACH1: AcCoA hydrolase
IDH1: NAD-dependent iso-citrate dehydrogenase
ENO1: Phosphopyruvate hydratase
GDH1: Glutamate dehydrogenase
MLS1: Malate synthase
HSP12: Heat shock protein12
SOD1: Cytosolic copper–zinc superoxide dismutase
FUM1: Fumarase
IDP1, IDP2: NADP-dependent iso-citrate dehydrogenase
CIT1: Citrate synthase
