# Supplementary material for: Metabolic switches from quiescence to growth in synchronized Saccharomyces cerevisiae
Source: Metabolomics. 2019 Aug 29;15(9):121. doi: 10.1007/s11306-019-1584-4 (PMC6715666; doi:10.1007/s11306-019-1584-4)
Supplement: Supplementary file 1 — Supplementary material 1 (DOCX 3333 kb) [file 11306_2019_1584_MOESM1_ESM.docx]

**Supplemental Figures and Tables**


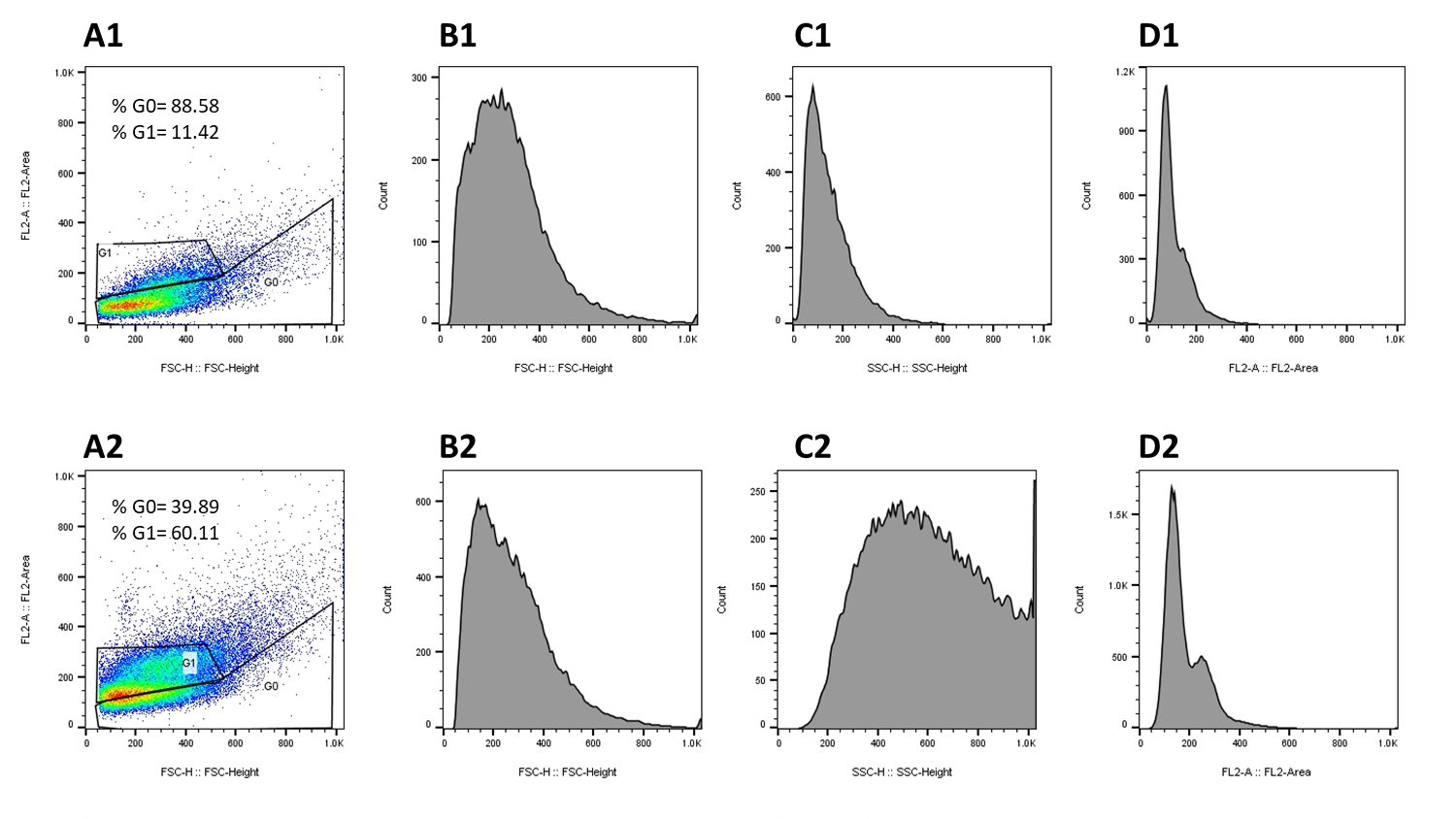


**Fig. S1.** Combinations of FSC-H, SSC-H and FL2-A scatters to compare 10 hours population starvation and after feeding for 10 minutes. Upper: 10 hours starvation (A1-D1); Bottom: (A2-D2) after 10 minutes feeding. A1 and A2 FL2-A versus FSC-H plots. B1 and B2. Forward-scattered light (FSC) fluorescence. C1 and C2. Side-scattered light (SSC) histograms. D1 and D2. Histogram of the PI-stained cells.


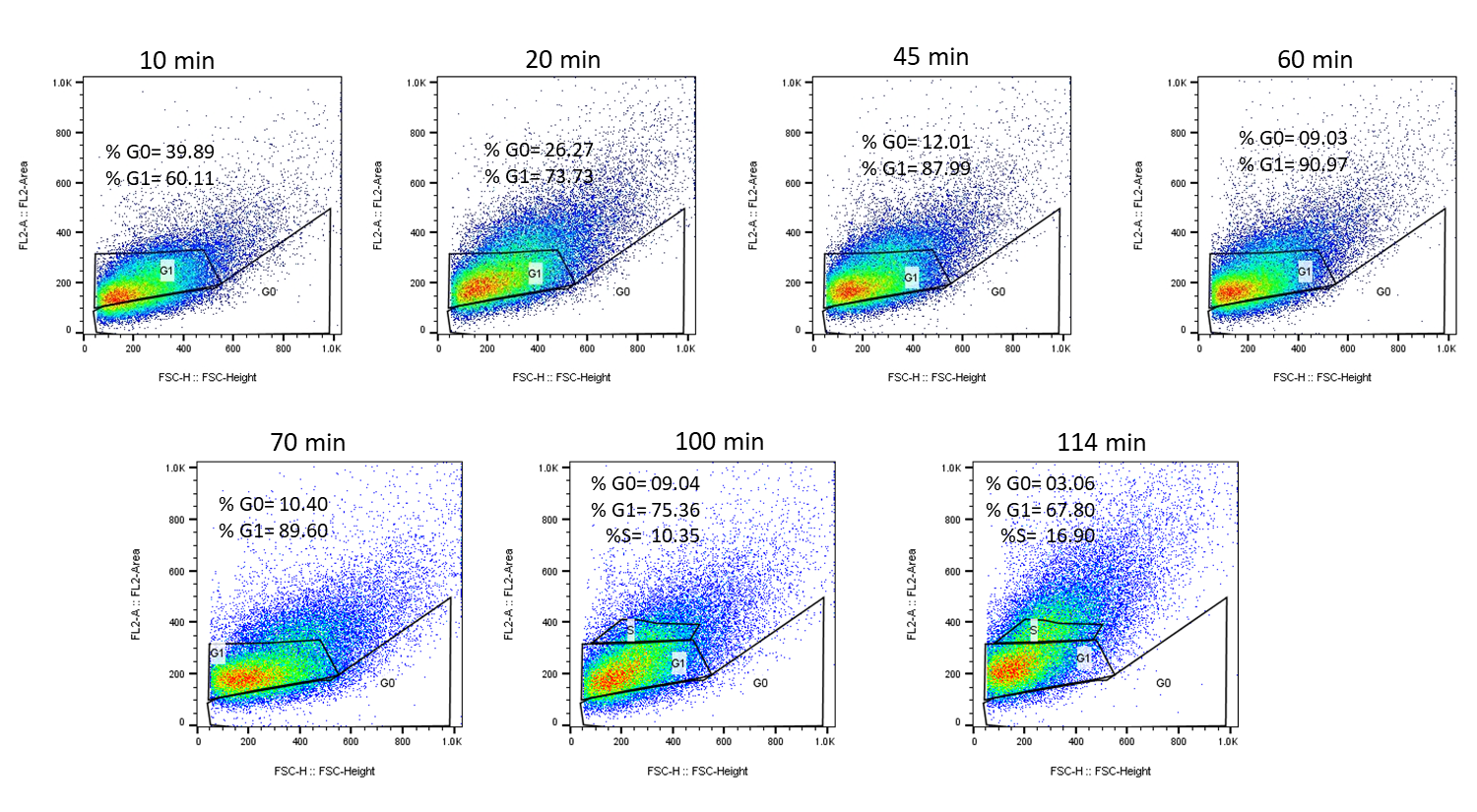


**Fig. S2.** Two dimensional representation (DNA staining signal/apparent size) used to determine gating parameters with PI showing samples of 10, 20, 45, 60, 70, 100 and 114 minutes. Gating was optimized for the whole growth experiment and is summarized by the representation of yeast populations at three typical stages. Flowjow software was used to reveal the percentage of cells in G0, G1 and S phase.


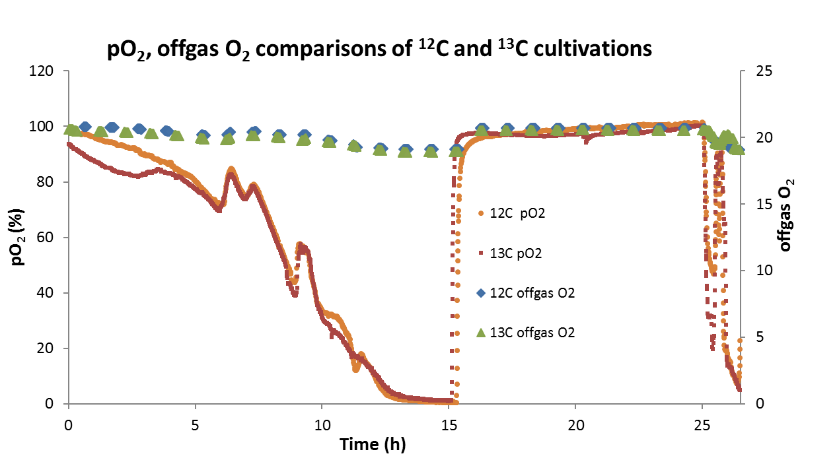

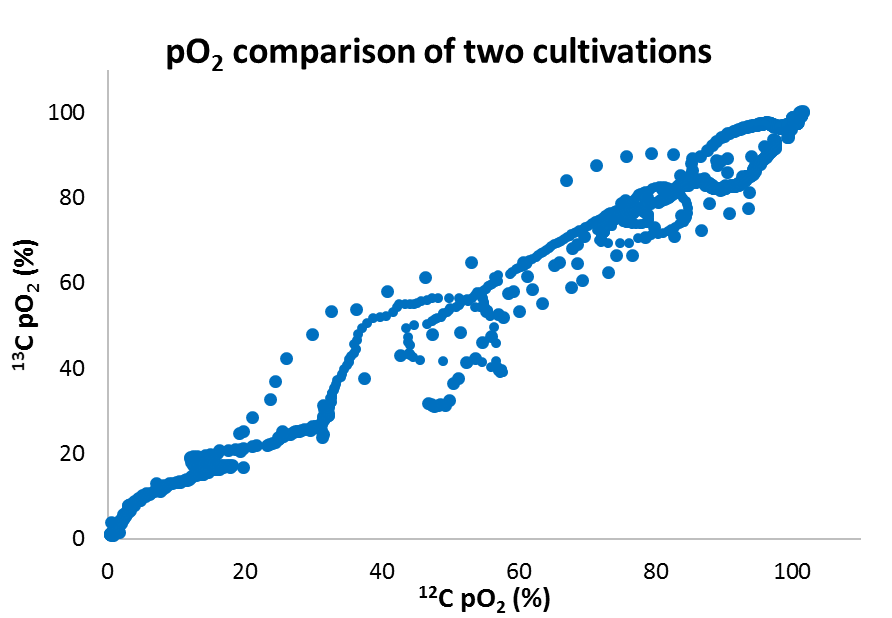


1. (B)

**Fig. S3.** pO_2_ and offgas O_2_ characteristic comparisons of the two cultivations: (A). pO_2_ and offgas O_2_ comparison of ^12^C and ^13^C experiments: 25 hours before and 2 hours after switch the feeding. (B). Correlation analysis of the pO_2_ profiles.

**B**

**A**

**Fig. S4.** (A) Biomass concentration measurements based on BugLab online measurements and predicted concentration based on a simple black-box piece-wise affine model. (B). Estimated specific growth rate (dots are breakpoints).

**A**

**B**

**C**

**Fig. S5.** (A). Extracellular glucose concentration after start of the continuous feeding. (B). Estimated specific glucose uptake rate after start of the feeding (normalized to biomass). (C). Glucose uptake rate as a function of the extracellular glucose concentration.


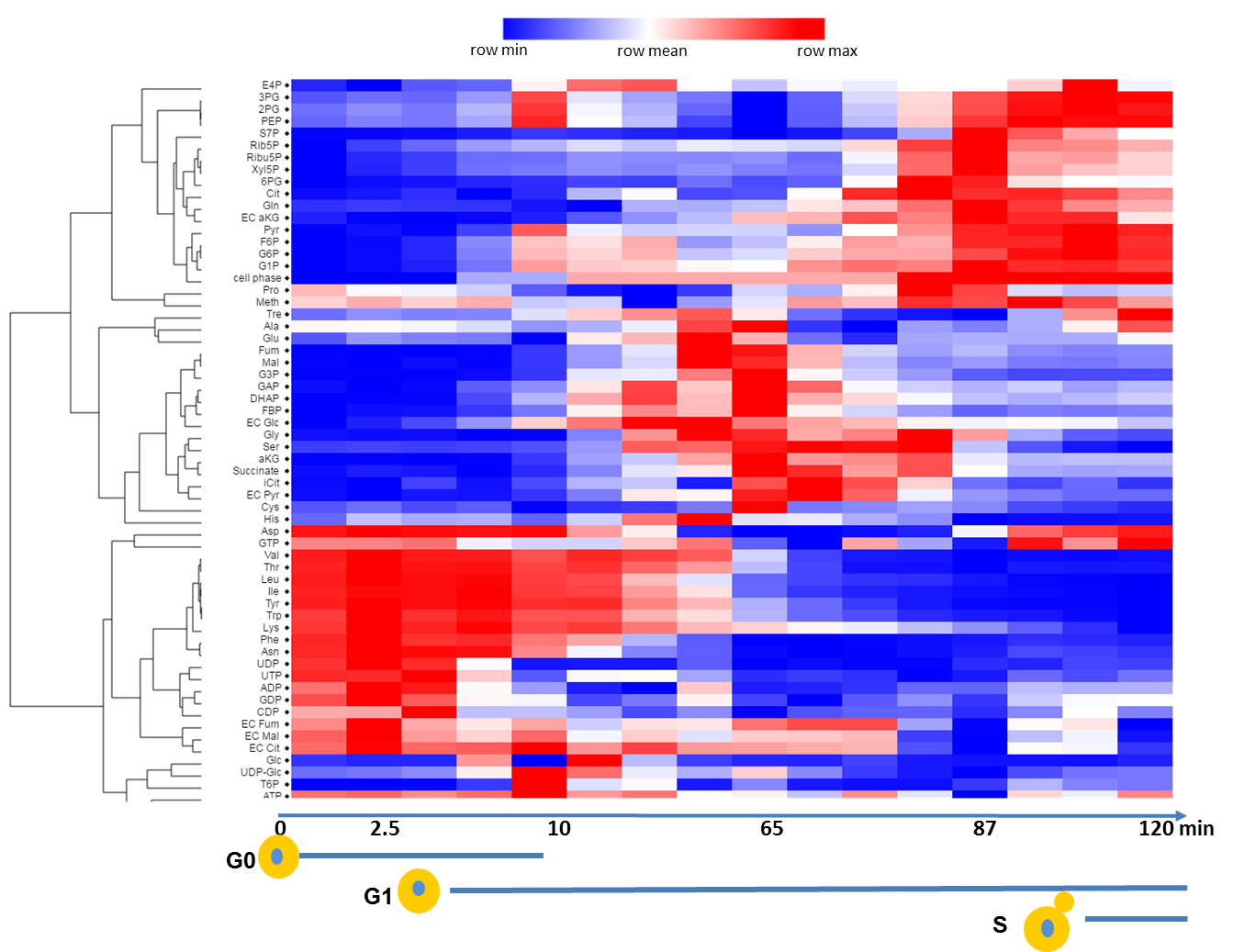


**A**

**B**

**Fig. S6.** Metabolic concentrations in G0 and during G0/G1/S transition. (A). Fold change (log2) of the intracellular concentrations between G0 (starved for 10 hours) and steady-state (D = 0.1 h^-1^). The two black dotted lines show the 1.2-fold changes (increased or decreased). (B). Hierarchical clustering of metabolite concentrations dynamics, including glycolytic, TCA cycle, PPP, and storage intermediates, nucleotides, amino acids, and extracellular metabolites. Blue to red is from the row minimum to row maximum.

**A**

**B**

**Fig. S7.** Dynamic response of other intracellular metabolite concentrations (black dot), enrichments (red cross), and simulations (lines) and nucleotides concentration. (A). glycolytic, TCA, PPP, storage pathway, and selected amino acids. *Glycogen enrichment measurement is not available. The black and the red lines are the best fit of the concentrations and enrichments measurements. (B). Nucleotides concentration as well as energy charge ($EC=\frac{\left[ATP\right]+1/2\left[ADP\right]}{\left[ATP\right]+\left[ADP\right]+\left[AMP\right]}$), AxP (the sum of all adenosine nucleotides) after start of feeding.

Flux at t=2.5 minutes Flux at t=10 minutes


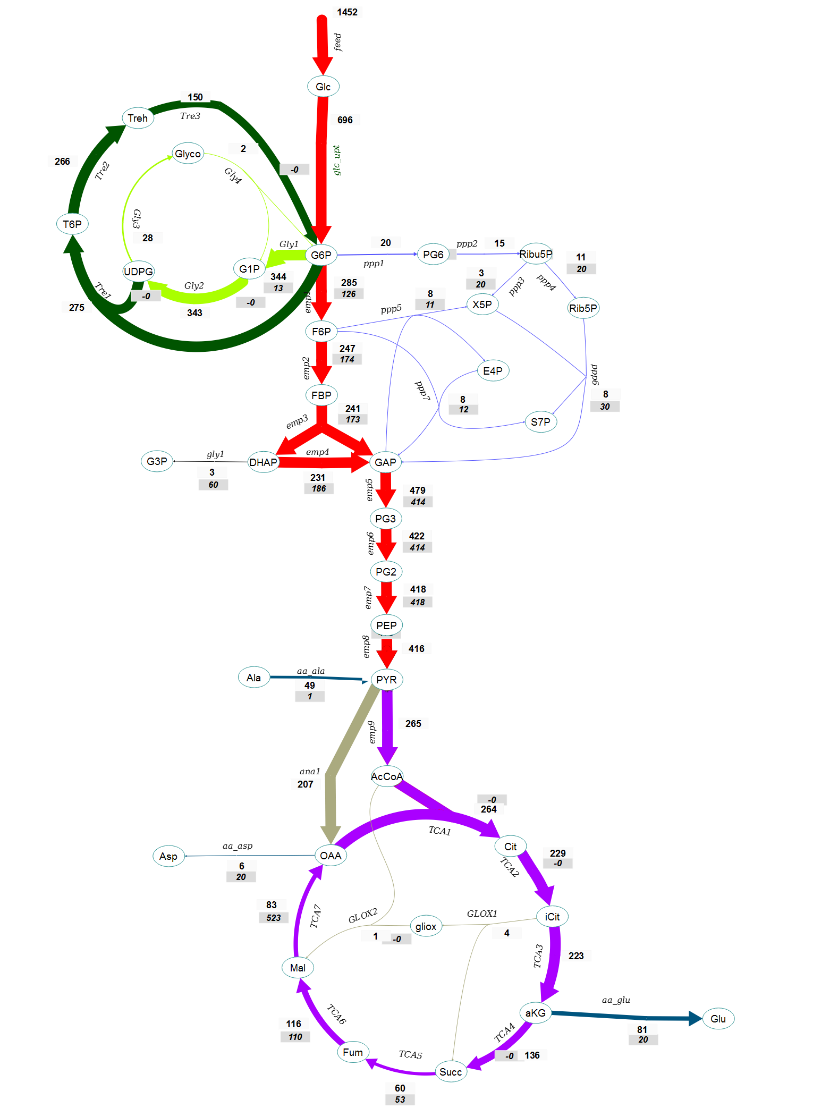

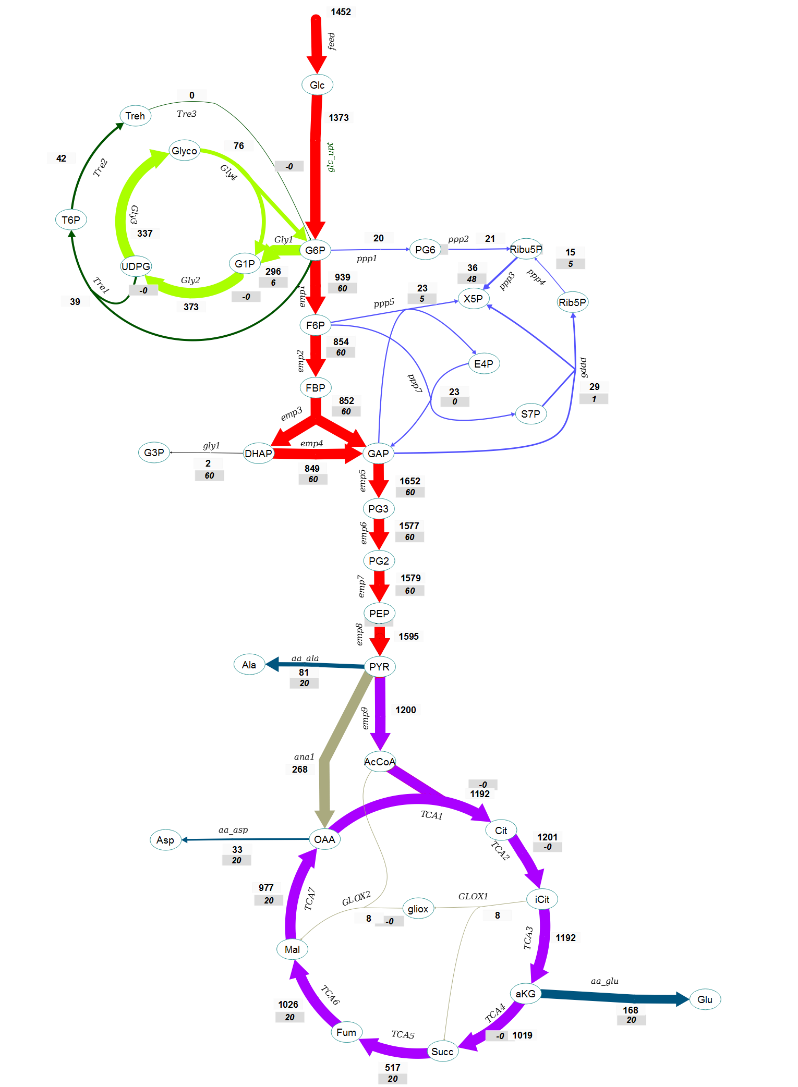


Flux at t=65 minutes Flux at t=87 minutes


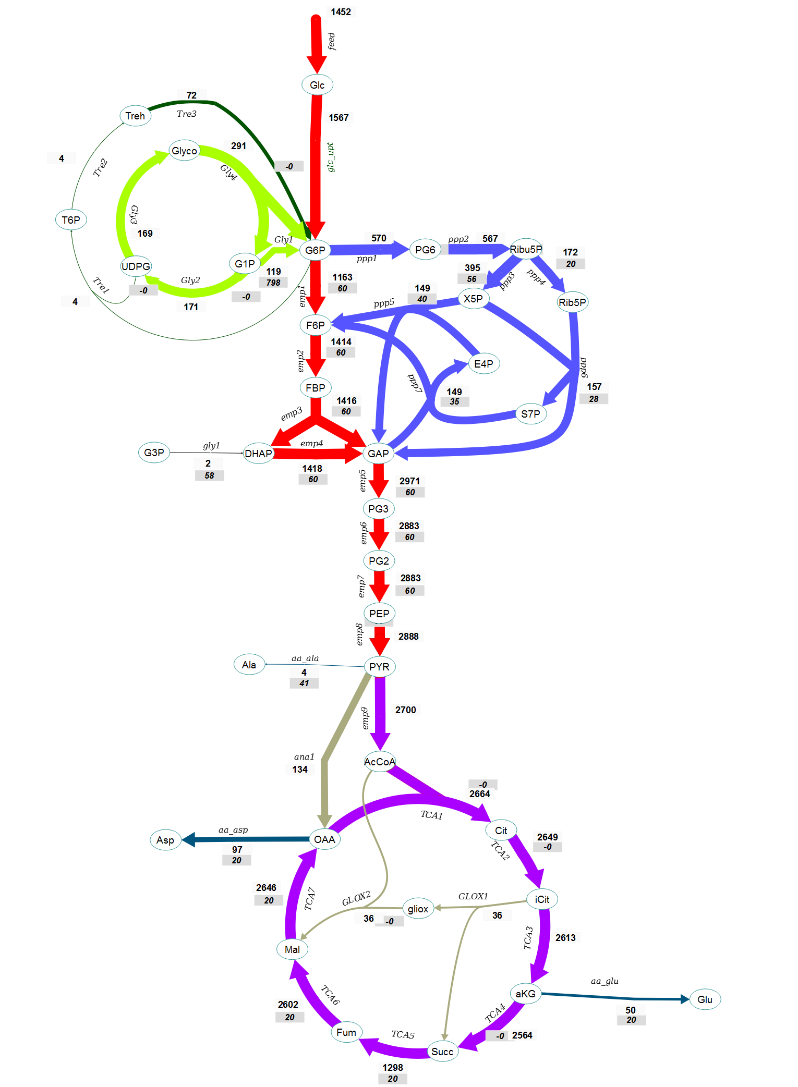

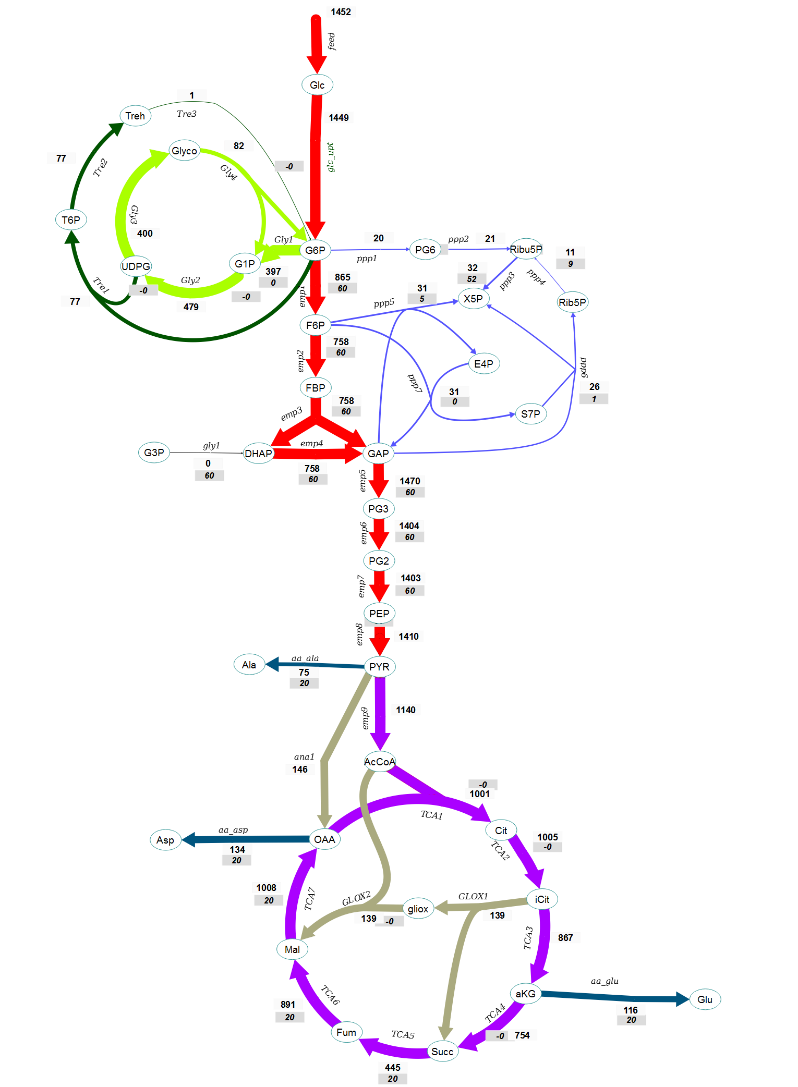


Flux at t=120 minutes


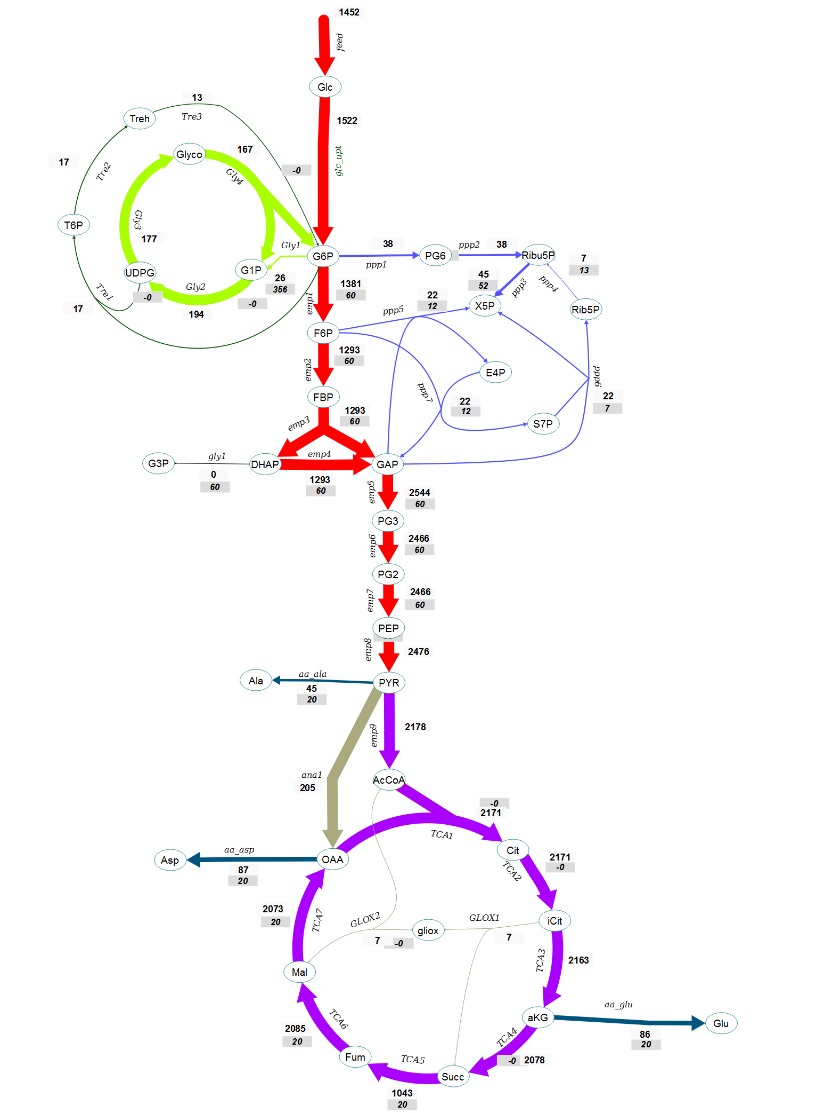


**Fig. S8.** Estimated flux values and flux map at t=2.5, 10, 65, 87, and 120 minutes. Flux values are given in μmol/gDW/h and listed in Table S2. Fluxes are categorized into 5 ranges (0-2, 2-10, 10-50, 50-500 and 500 above). Within each flux range, the width of the arrow corresponds to the net flux values with a maximal thickness of 20 pixels and a minimum value of 1. The net flux and backward flux (for reversible reaction) values are shown.

min:0 30 60 90 120 SS

ICL1

MLS1

FBP1

PYC1

PCK1

0

6.66

12.50

Glycolysis

Storage

PPP

TCA

'de novo'

purine

nucleotide biosynthetic

tRNA

ligase

37S

ribosomal

protein

60S

ribosomal

protein

40S

ribosomal

protein

54S

ribosomal

protein

Chorismate

gluconeogenic

glyoxylate

amino acids

min:0 30 60 90 120 SS

**Fig. S9.** Heat map of the protein levels grouped in selected functional categories during G0/G1 transition. Protein levels were normalized to the steady-state protein levels (last column). ICL1, MLS1, FBP1, PYC1 and PCK1 were not detected in steady-state samples. Therefore, their related changes were compared with a different scale (bottom right).

**Table S1.** Stoichiometric reactions for the metabolic network including the atom transition with each reaction. Unidirectional fluxes use “>” as the reaction arrow while bidirectional fluxes use “<>” as the reaction arrow. T6P was shown separately as trehalose c1-c6 and c7-c12.

| Flux: reaction | Atom transition |
| --- | --- |
| feed: feedA > Glc_ec | #abcdef > #abcdef |
| glc_upt: Glc_ec > G6P | #abcdef > #abcdef |
| Tre1: G6P + UDPG > T6P(c1-c6) +T6P(c7-c12) | #abcdef+ #ABCDEF> #abcdef+ #ABCDEF |
| Tre2: T6P(c1-c6) +T6P(c7-c12) <> Treh | #abcdef + #ABCDEF> #abcdefABCDEF |
| Tre3: Treh > G6P + G6P | #abcdefABCDEF> #abcdef + #ABCDEF |
| Gly1: G6P <> G1P | #abcdef > #abcdef |
| Gly2: G1P > UDPG | #abcdef > #abcdef |
| Gly3: UDPG > Glyco | #abcdef > #abcdef |
| Gly4: Glyco > G1P | #abcdef > #abcdef |
| emp1: G6P <> F6P | #abcdef > #abcdef |
| emp2: F6P <> FBP | #ABCDEF> #ABCDEF |
| emp3: FBP <> DHAP+GAP | #ABCDEF> #CBA+#DEF |
| emp4: DHAP <> GAP | #cba > #abc |
| emp5: GAP <> PG3 | #abc > #abc |
| emp6: PG3 <> PG2 | #abc > #abc |
| emp7: PG2 <> PEP | #abc > #abc |
| emp8: PEP > PYR | #abc > #abc |
| gly1: DHAP <> G3P | #abc > #abc |
| ppp1: G6P > PG6 | #abcdef > #abcdef |
| ppp2:PG6 > CO2 + Ribu5P | #abcdef > #a + #bcdef |
| ppp3: Ribu5P <> X5P | #abcde > #abcde |
| ppp4: Ribu5P <> Rib5P | #abcde > #abcde |
| ppp5: X5P + E4P <> GAP + F6P | #ABCDE + #abcd > #CDE + #ABabcd |
| ppp6: X5P + Rib5P <> S7P + GAP | #ABCDE + #abcde > #ABabcde + #CDE |
| ppp7: GAP + S7P <> E4P + F6P | #ABC + #abcdefg> #defg + #abcABC |
| emp9: PYR > AcCoA + CO2 | #abc > #bc + #a |
| TCA1: AcCoA + OAA > Cit | #AB + #abcd > #dcbaBA |
| TCA2: Cit > iCit | #ABCDEF > #ABCDEF |
| TCA3: iCit > aKG + CO2 | #ABCDEF > #ABCEF + #D |
| TCA4: aKG > Succ + CO2 | #ABCDE > #BCDE + #A |
| TCA5: Succ + Succ <> Fum + Fum | #ABCD + #abcd > #ABCD + #dcba |
| TCA6: Fum <> Mal | #ABCD > #ABCD |
| TCA7: Mal <> OAA | #ABCD > #ABCD |
| GLOX1: iCit > glyox + Succ | #ABCDEF >#AB + #DCEF |
| GLOX2: AcCoA + glyox > Mal | #AB + #ab > #ABab |
| aa_glu: aKG <> Glu | #ABCDE > #ABCDE |
| aa_asp: OAA <> Asp | #ABCD > #ABCD |
| aa_ala: PYR <> Ala | #abc > #abc |
| ana1: PYR + CO2 > OAA | #abc + #d > #abcd |
| ana2: OAA > PEP + CO2 | #abcd > #abc + #d |
| ana3: Mal > PYR + CO2 | #ABCD > #ABC + #D |
| BIO1: G6P > G6P_bm | # abcdef > # abcdef |
| BIO2: F6P > F6P_bm | # abcdef > # abcdef |
| BIO3: PG3> PG3_bm | # abc > # abc |
| BIO4: G3P > G3P_bm | # abc > # abc |
| BIO5: PEP > PEP_bm | #abc > #abc |
| BIO6: E4P > E4P_bm | # abcd > # abcd |
| BIO7: Rib5P > Rib5P_bm | # abcde > # abcde |
| BIO8: Treh > Treh_bm | #abcdef > #abcdef |
| BIO9: Glyco > Glyco_bm | #abcdef > #abcdef |
| mRNA_exchange: Rib5P <> mRNA | #abcde > #abcde |

**Table S2.** Best flux estimates at t=2.5, 10, 65, 87, and 120 minutes (values are given in μmol/gDW/h), and the corresponding enzymes level was normalized to steady-state level. “–”: below the limit of detection. “– – ”: multi-enzymes. “*”: when the steady-state enzyme level was below the limit of detection, they were normalized to ^13^C internal standard. Bold: interesered changes.

| Reaction | Enzyme | Net flux (μmol/gDW/h) | | | | | | Protein (normalized to steady-state level) | | | | | | |  |
| --- | --- | --- | --- | --- | --- | --- | --- | --- | --- | --- | --- | --- | --- | --- | --- |
|  |  | t=0 | t=2.5 | t=10 | t=65 | t=87 | t=120 |  | t=0 | t=30 | t=60 | t=90 | t=120 | SS | |
| Feed | - | 0 | 1452.1 | 1452.1 | 1452.1 | 1452.1 | 1452.1 |  | - | - | - | - | - | - | |
| glc_upt | Hexokinase | 0 | 696.4 | 1372.6 | 1567.5 | 1449.3 | 1521.6 | HXK1 | **0.41±0.15** | **0.49±0.15** | **0.41±0.15** | **0.46±0.14** | **0.65±0.15** | 1±0.21 | |
|  |  |  |  |  |  |  |  | HXK2 | 1.06±0.22 | 1.13±0.25 | 1.23±0.34 | 1.36±0.45 | 1.41±0.41 | 1±0.41 | |
|  |  |  |  |  |  |  |  | GLK1 | **0.68±0.24** | 0.80±0.16 | 0.83±0.17 | 0.78±0.18 | 0.83±0.16 | 1±0.14 | |
| Tre1 | Trehalose-P synthase | 0 | 275.4±15.3 | 39.5±5.8 | 4.3±9.3 | 77.4±10.2 | 16.5±15.7 | TPS1 | **1.32±0.15** | **1.39±0.23** | **1.39±0.23** | 1.14±0.22 | 1.18±0.23 | 1±0.13 | |
| Tre2 | Trehalose-phosphatase | 0 | 266.4±20.2 | 41.5±6.2 | 3.7±10.7 | 77.3±40.2 | 16.5±72.2 | TPS2 | 1.38±0.22 | **2.03±0.62** | **1.51±0.20** | 1.42±0.20 | **1.71±0.20** | 1±0.33 | |
| Tre3 | Trehalase | 0 | 150.0±15.3 | 0.0±5.8 | 72.4±9.3 | 0.5±10.2 | 13.3±15.7 | ATH1  NTH1 | - | - | - | - | - | - | |
| Gly1 | P-glucomutase | 0 | 344.1±434.6 | 295.9±258.8 | -119.4±193.4 | 397.4±200.6 | 26.2±187.9 | PGM2 | 0.98±0.27 | 1.08±0.26 | 1.05±0.33 | 0.99±0.28 | 1.25±0.47 | 1±0.30 | |
| Gly2 | UTP-G1P uridylyltransferase | 0 | 343.2±24.6 | 372.5±62.3 | 171.1±47.8 | 478.8±52.0 | 193.6±66.2 | UGP1 | 0.8±0.10 | 0.79±0.18 | 0.72±0.03 | 0.91±0.21 | 1.04±0.09 | - | |
| Gly3 | Glycogenin glucosyltransferase | 0 | 28.1±28.0 | 337.5±62.4 | 169.4±49.8 | 400.1±54.2 | 177.1±73.6 | GLG1 | **2.36±0.20** | **1.53±0.20** | **1.52±0.20** | **1.41±0.20** | - | - | |
|  | Glycogen synthase |  |  |  |  |  |  | GSY1 | **0.61±0.03** | **0.71±0.00** | **0.56±0.18** | **0.43±0.12** | **0.52±0.12** | 1±0.20 | |
|  |  |  |  |  |  |  |  | GSY2 | 0.82±0.20 | **0.64±0.15** | **0.69±0.20** | 0.82±0.20 | 0.76±0.00 | 1±0.02 | |
|  | Glucan branching enzyme |  |  |  |  |  |  | GLC3 | 0.83±0.20 | **0.53±0.20** | **0.60±0.05** | **0.60±0.20** | **0.59±0.20** | 1±0.06 | |
| Gly4 | Glycogen phosphorylase | 0 | 1.8±28.0 | 76.3±62.4 | 290.8±49.8 | 81.5±54.2 | 167.4±73.6 | GPH1 | **0.55±0.04** | 0.83±0.10 | **0.62±0.11** | **0.6±0.10** | **0.65±0.10** | 1±0.13 | |
| emp1 | G6P isomerase | 0 | 284.7±4.2 | 939.5±12.7 | 1163.2±159.1 | 864.8±8.6 | 1380.8±24.5 | PGI1 | 1.22±0.23 | 1.14±0.43 | 1.38±0.19 | 1.15±0.53 | 1.23±0.25 | 1±0.25 | |
| emp2 | P-fructokinase | 0 | 246.8±1.4 | 854.1±4.2 | 1414.0±53.1 | 757.7±2.9 | 1293.0±8.2 | PFK1 | 1.15±0.25 | 1.19±0.27 | 1.28±0.26 | 1.31±0.24 | 1.25±0.29 | 1±0.28 | |
|  |  |  |  |  |  |  |  | PFK2 | 1.09±0.32 | 1.23±0.40 | 1.2±0.37 | 1.17±0.32 | 1.06±0.41 | 1±0.19 | |
|  | Fructose bisphosphatase |  |  |  |  |  |  | FBP1* | **12.5±2.0** | **11.49±3.49** | **9.35±2.0** | **9.71±2.0** | **8.26±0.20** | - | |
| emp3 | Fructose-1,6P aldolase | 0 | 241.0±1.4 | 852.2±4.2 | 1415.8±53.1 | 757.7±2.9 | 1293.0±8.2 | FBA1 | 0.84±0.29 | 0.85±0.31 | 0.85±0.36 | 0.92±0.31 | 0.88±0.21 | 1±0.16 | |
| emp4 | Triose-P isomerase | 0 | 231.0±1.4 | 849.3±4.2 | 1418.2±53.1 | 758.3±2.9 | 1293.0±8.2 | TPI1 | 1.0±0.29 | 1.2±0.26 | 1.2±0.32 | 1.13±0.26 | 1.14±0.28 | 1±0.22 | |
| emp5 | Glyceraldehyde-3P dehydrogenase | 0 | 479.4±1.4 | 1652.0±4.2 | 2970.6±53.1 | 1470.2±2.9 | 2543.9±8.2 | TDH1 | **0.62±0.19** | **0.53±0.14** | **0.44±0.1** | **0.5±0.18** | **0.5±0.03** | 1±0.35 | |
|  |  |  |  |  |  |  |  | TDH2 | 1.16±0.65 | 1.43±0.6 | 1.39±0.94 | 1±0.49 | 1.12±0.55 | 1±0.55 | |
|  |  |  |  |  |  |  |  | TDH3 | 0.89±0.26 | 0.96±0.35 | 0.94±0.23 | 0.84±0.29 | 0.79±0.21 | 1±0.22 | |
|  | 3P-glycerate kinase |  |  |  |  |  |  | PGK1 | 0.9±0.30 | 0.88±0.22 | 0.88±0.21 | 0.82±0.21 | 0.78±0.15 | 1±0.34 | |
| emp6 | P-glycerate mutase | 0 | 422.2±1.4 | 1577.2±4.2 | 2883.5±53.1 | 1403.5±2.9 | 2465.9±8.2 | GPM1 | 0.94±0.26 | 1.04±0.26 | 0.89±0.51 | 1±0.34 | 0.77±0.17 | 1±0.16 | |
|  |  |  |  |  |  |  |  | GPM2,3 | - | - | - | - | - | - | |
| emp7 | Enolase | 0 | 417.8±1.4 | 1578.6±4.2 | 2882.6±53.1 | 1403.0±2.9 | 2465.9±8.2 | ENO1 | **0.48±0.06** | **0.54±0.25** | **0.55±0.18** | **0.38±0.13** | **0.48±0.11** | 1±0.26 | |
|  |  |  |  |  |  |  |  | ENO2 | 1.13±0.46 | 1.1±0.43 | 1±0.37 | 1.02±0.43 | 0.99±0.38 | 1±0.19 | |
| emp8 | Pyruvate kinase | 0 | 416.2±200.2 | 1595.0±199.4 | 2888.0±532.0 | 1410.3±658.5 | 2475.6±2136.8 | PYK1 | 1.34±0.36 | 1.47±0.27 | 1.32±0.26 | 1.3±0.22 | 1.02±0.56 | 1±0.25 | |
|  |  |  |  |  |  |  |  | PYK2 | - | - | - | - | - | - | |
| emp9 | Pyruvate dehydrogenase | 0 | 265.2±9.3 | 1200.0±18.1 | 2700.0±125.0 | 1140.0±782.0 | 2178.0±2441.8 | PDA1 | 1.32±0.36 | 1.30±0.26 | 1.39±0.20 | 1.63±0.23 | 1.28±0.25 | 1±0.35 | |
|  |  |  |  |  |  |  |  | PDB1 | 1.08±0.24 | 1.12±0.15 | 1.08±0.25 | 0.96±0.11 | 0.94±0.30 | 1±0.30 | |
|  | Dihydrolipoyl dehydrogenase |  |  |  |  |  |  | LAT1 | 1.07±0.39 | 1.21±0.15 | 1.22±0.27 | 1.07±0.46 | 1.08±0.34 | 1±0.16 | |
|  |  |  |  |  |  |  |  | LPD1 | 1.97±0.27 | 2.68±0.11 | 2.41±0.20 | 1.89±0.31 | 1.98±0.30 | 1±0.30 | |
| gly1 | glycerol-3P dehydrogenase | 0 | 2.6±0 | 1.9±0 | -1.5±0 | -0.2±0 | 0.0±0 | GPD1 | 1.22±0.27 | 1.27±0.12 | 1.2±0.12 | 1.4±0.22 | 1.55±0.24 | 1±0.19 | |
|  |  |  |  |  |  |  |  | GPD2 | - | - | - | - | - | - | |
| ppp1 | G6P-dehydrogenase | 0 | 19.6±4.2 | 19.6±12.7 | 570.0±159.2 | 19.6±8.1 | 37.6±24.5 | ZWF1 | 1.22±0.34 | 1.15±0.40 | 1.07±0.36 | 1.28±0.29 | 1.26±0.39 | 1±0.21 | |
| ppp2 | 6P-gluconate dehydrogenase | 0 | 14.9±4.2 | 21.4±12.7 | 567.0±159.2 | 20.9±8.1 | 37.6±24.5 | GND1 | **0.55±0.18** | **0.64±0.13** | **0.64±0.08** | 0.76±0.17 | 0.90±0.20 | 1±0.25 | |
| ppp3 | Ribulose-P epimerase | 0 | 3.1±2.8 | 36.4±8.5 | 394.6±106.1 | 31.8±5.8 | 44.7±16.4 | RPE1 | **1.46±0.20** | **1.52±0.16** | **1.65±0.35** | 1.17±0.00 | 1.47±0.46 | 1±0.20 | |
| ppp4 | Ribulose-P 3-epimerase | 0 | -8.2±1.4 | -22.5±4.2 | 148.6±53.1 | -31.3±2.9 | -22.1±8.2 | RKI1* | **3.70**±0.08 | **3.62**±0.03 | **3.39**±0.06 | **3.37**±0.04 | **3.57**±0.02 | - | |
| ppp5 | Transketolase | 0 | 7.9±1.4 | -29.4±4.2 | 156.5±53.1 | -25.8±2.9 | -22.1±8.2 | TKL1 | 1.03±0.29 | 1.09±0.57 | 1.24±0.29 | 1.45±0.41 | 1.3±0.28 | 1±0.30 | |
| ppp6 | Transketolase | 0 | 7.9±1.4 | -29.4±4.2 | 156.5±53.1 | -25.8±2.9 | -22.1±8.2 | TKL2 | - | - | - | - | - | - | |
| ppp7 | Transaldolase | 0 | 8.1±1.4 | -22.5±4.2 | 148.6±53.1 | -31.3±2.9 | -22.1±8.2 | TAL1 | 0.78±0.27 | 0.84±0.35 | 0.73±0.32 | 0.81±0.34 | 0.9±0.30 | 1±0.21 | |
| TCA1 | Citrate synthase | 0 | 264.5±1.4 | 1191.6±4.2 | 2663.9±53.1 | 1000.6±2.9 | 2170.5±8.2 | CIT1 | **2.61±0.17** | **2.82±0.09** | **2.34±0.15** | **2.53±0.15** | **2.56±0.13** | 1±0.40 | |
| TCA2 | Aconitate hydratase | 0 | 228.8±1.4 | 1200.7±4.2 | 2649.2±53.1 | 1004.6±2.9 | 2170.5±8.2 | ACO1 | **1.82±0.13** | **1.94±0.12** | **1.77±0.15** | **1.67±0.16** | **1.50±0.17** | 1±0.14 | |
|  |  |  |  |  |  |  |  | ACO2 | 0.76±0.31 | 0.66±0.32 | 0.69±0.22 | 0.79±0.41 | 0.79±0.14 | 1±0.06 | |
| TCA3 | Iso-citrate dehydrogenase | 0 | 222.5±9.1 | 1191.9±12.8 | 2612.6±71.0 | 867.0±781.9 | 2163.1±2442.1 | IDH1 | **3.06±0.13** | **3.29±0.08** | **2.78±0.08** | **2.55±0.18** | **2.62±0.10** | 1±0.29 | |
|  |  |  |  |  |  |  |  | IDH2 | **2.71±0.07** | **2.67±0.09** | **2.56±0.08** | **2.55±0.05** | **2.20±0.05** | 1±0.22 | |
|  |  |  |  |  |  |  |  | IDP1 | **1.94±0.19** | **1.90±0.13** | **1.79±0.09** | **1.93±0.14** | **1.68±0.16** | 1±0.38 | |
|  |  |  |  |  |  |  |  | IDP2 | **2.68±0.13** | **2.63±0.16** | **2.63±0.14** | **2.38±0.15** | **2.17±0.23** | 1±0.46 | |
| TCA4 | 2-oxoglutarate dehydrogenase | 0 | 136.5±9.1 | 1019.0±12.8 | 2564.0±71.0 | 753.8±781.9 | 2077.6±2442.1 | KGD1 | **2.75±0.12** | **2.83±0.13** | **2.66±0.12** | **2.63±0.17** | **2.18±0.14** | 1±0.45 | |
|  | Succinyl-CoA ligase |  |  |  |  |  |  | LSC1 | 0.90±0.06 | 0.93±0.17 | 0.91±0.17 | 0.90±0.12 | 0.83±0.36 | 1±0.21 | |
|  |  |  |  |  |  |  |  | LSC2 | 1.02±0.40 | 1.44±0.16 | 1.17±0.50 | 1.46±0.01 | 1.0±0.45 | 1±0.10 | |
|  | Dihydrolipoyl dehydrogenase |  |  |  |  |  |  | LPD1 | **1.97±0.2** | **2.68±0.11** | **2.41±0.20** | **1.89±0.31** | **1.98±0.30** | 1±0.61 | |
| TCA5 | Succinate dehydrogenase | 0 | 119.4±0.7 | 1033.8±2.1 | 2595.8±26.5 | 890.8±1.4 | 2085.0±4.1 | SDH1 | **2.51±0.09** | **2.49±0.16** | **2.48±0.13** | **2.22±0.16** | **2.08±0.10** | 1±0.34 | |
|  |  |  |  |  |  |  |  | SDH2 | **2.08±0.18** | **2.64±0.09** | **2.64±0.18** | **2.61±0.07** | **1.81±0.20** | 1±0.24 | |
|  |  |  |  |  |  |  |  | SDH3 | **2.12±0.03** | **2.80±0.04** | **2.38±0.17** | **1.51±0.20** | **2.02±0.22** | 1±0.20 | |
|  |  |  |  |  |  |  |  | SDH4 | 1.05±0.20 | 1.13±0.20 | 1.16±0.20 | 1.15±0.20 | 1.65±0.20 | 1±0.20 | |
| TCA6 | Fumarate hydratase | 0 | 115.9±1.4 | 1025.7±4.2 | 2601.6±53.1 | 890.6±2.9 | 2085.0±8.2 | FUM1 | **2.17±0.31** | **2.38±0.23** | **2.09±0.27** | **2.11±0.25** | **2.12±0.33** | 1±0.24 | |
| TCA7 | Malate dehydrogenase | 0 | 82.8±204.7 | 976.5±43.5 | 2646.5±603.0 | 1008.2±928.7 | 2072.9±2988.0 | MDH1 | **1.96±0.19** | **1.88±0.18** | **1.93±0.19** | **1.80±0.17** | **2.05±0.19** | 1±0.31 | |
| GLOX1 | Iso-citrate lyase | 0 | 4.3±9.1 | 8.0±15.1 | 36.1±86.7 | 139.3±782.0 | 7.5±2441.4 | ICL1 | **8.66±0.08** | **9.95±0.07** | **7.15±0.09** | **9.56±0.05** | **8.56±0.07** | 1±0.25 | |
| GLOX2 | Malate synthase | 0 | 0.7±9.1 | 8.4±15.1 | 36.1±86.7 | 139.4±782.0 | 7.5±2441.4 | MLS1 | **6.91±0.13** | **11.24±0.07** | **8.35±0.12** | **9.85±0.07** | **12.01±0.04** | 1±0.16 | |
| aa_glu | Glutamate dehydrogenase | 0 | 81.0±113.5 | 168.5±14.9 | 49.7±34.6 | 115.7±15.4 | 85.5±52.1 | GDH1 | **0.26±0.44** | **0.27±0.30** | **0.34±0.36** | **0.51±0.13** | **0.45±0.22** | 1±0.11 | |
|  |  |  |  |  |  |  |  | GDH2 | **1.75±0.26** | **2.11±0.18** | **1.92±0.27** | **1.65±0.18** | **1.55±0.37** | 1±0.46 | |
| aa_asp | Aspartate aminotransferase | 0 | 5.7±76.7 | 32.8±12.6 | 97.1±37.9 | 134.1±63.2 | 87.3±62.6 | AAT2 | **2.59±0.12** | **2.58±0.06** | **2.55±0.09** | **2.63±0.11** | **1.99±0.09** | 1±0.05 | |
| aa_ala | Alanine transaminase | 0 | -49.1±53.6 | 81.0±11.8 | 4.2±20.1 | 74.8±17.3 | 44.6±26.0 | ALT1 | - | - | - | - | - | - | |
| ana1 | Pyruvate carboxylase | 0 | 207.0±13.7 | 267.6±199.7 | 134.1±309.7 | 146.2±720.6 | 204.6±2221.9 | PYC1* | **2.71±0.07** | **2.86±0.1** | **2.63±0.2** | **2.75±0.17** | **2.36±0.09** | **-** | |
| ana2 | PEP carboxykinase | 0 | 19.6±200.0 | 19.6±199.3 | 19.6±525.3 | 19.6±658.4 | 19.6±2136.3 | PCK1* | **4.02±0.19** | **5.99±0.1** | **12.5±0.08** | **11.9±0.06** | **6.45±0.15** | **-** | |
| ana3 | -- | 0 | 19.6±204.4 | 19.6±39.4 | 19.6±575.3 | 19.6±820.9 | 19.6±2645.0 | - | - | - | - | - | - | - | |
| BIO1 | -- | 0 | 21.03 | 21.0 | 21.0 | 21.1 | 21.0 | - | - | - | - | - | - | - | |
| BIO2 | -- | 0 | 21.0 | 21.0 | 20.96 | 21.01 | 21.0 | - | - | - | - | - | - | - | |
| BIO3 | -- | 0 | 21.03 | 21.0 | 21.01 | 21.01 | 20.98 | - | - | - | - | - | - | - | |
| BIO4 | -- | 0 | 20.97 | 21.03 | 20.98 | 20.99 | 20.97 | - | - | - | - | - | - | - | |
| BIO5 | -- | 0 | 20.96 | 20.99 | 20.96 | 21.01 | 21.0 | - | - | - | - | - | - | - | |
| BIO6 | -- | 0 | 21.04 | 20.96 | 21.0 | 20.99 | 20.99 | - | - | - | - | - | - | - | |
| BIO7 | -- | 0 | 20.98 | 21.03 | 21.01 | 21.0 | 21.0 | - | - | - | - | - | - | - | |
| BIO8 | -- | 0 | 21.03 | 20.99 | 21.02 | 21.02 | 21.03 | - | - | - | - | - | - | - | |
| BIO9 | -- | 0 | 20.98 | 20.98 | 21.0 | 20.98 | 21.01 | - | - | - | - | - | - | - | |
